# Subclassification of Small Cell Lung Cancer Based on Gene Expression Signatures and Machine Learning

**DOI:** 10.1158/2767-9764.CRC-25-0512

**Published:** 2026-03-12

**Authors:** Nicole Kiedanski, Julian Kreis, Lucia Spangenberg, Eike Staub

**Affiliations:** 1 Oncology Data Science, The Healthcare Business of Merck KGaA, Darmstadt, Germany.; 2 Bioinformatics Unit, Institut Pasteur de Montevideo, Montevideo, Uruguay.; 3 Departamento Básico de Medicina, Hospital de Clínicas, Facultad de Medicina (UDELAR), Montevideo, Uruguay.

## Abstract

Small cell lung cancer (SCLC) is frequently subdivided into four molecular subtypes according to the activity of key transcription factors (TF): *NEUROD1*, *ASCL1*, *POU2F3*, and *YAP1* (NAPY). There is no consensus on the diagnostic procedures to determine these subtypes. Downstream transcriptional programs of the four TFs could play an important role in the development of an advanced SCLC subtyping approach. We analyzed transcriptomic and genomic sequencing data from a novel cohort of 460 real-world patients with SCLC. We extracted gene expression signatures specific for the four TFs and used them as features to train machine learning (ML) models to predict SCLC-NAPY subtypes in a nested cross-validation approach. Our ML model for transcriptional programs downstream of the four NAPY TFs predicted the NAPY subtypes at an average accuracy of ∼90% in clinical SCLC samples and cell lines. We assessed genomic alterations and our compendium of cancer pathway signatures RosettaSX for subtype-specific signals in our SCLC cohort. Survival analyses of an extensive-stage SCLC subset revealed significant prognostic differences and predictive capacities for several molecular phenotypes. We propose a diagnostic algorithm for NAPY classification which demands that high expression of a NAPY TF is matched by a high signal of its downstream expression signature, thereby providing functional robustness to NAPY class calls compared with schemes that rely solely on the expression of TFs. Based on this NAPY consensus classification of our SCLCs, we describe the redetection of known and identification of novel associations of molecular and clinical patters across the four major molecular subtypes.

**Significance:**

Based on analysis of our novel real-word SCLC cohort, we extend the methods space for SCLC diagnosis by developing four downstream transcriptional programs linked to the key TFs that we used for ML-based NAPY classification. By combining evidence from TFs and their downstream signatures, we add functional robustness to current classification schemes. Furthermore, we describe distinct molecular and clinical patters observed across our NAPY subtypes.

## Introduction

Small cell lung cancer (SCLC), notorious for its aggressive behavior and rapid progression, poses a substantial challenge in thoracic oncology. It comprises approximately 15% of all lung cancer cases, with a median survival duration of <2 years for patients with early-stage disease and approximately 1 year for patients with metastatic disease (1). The existence of distinct molecular subtypes of SCLC is widely accepted, with the support of more than two decades of research. A classification referring to the four major distinct subtypes, based on the expression of key transcription factors (TF), namely *NEUROD1*, *ASCL1*, *POU2F3*, and *YAP1*, has gain broad recognition among investigations in the field and has been referred to in the recent World Health Organization classification of thoracic tumors (2, 3).

Recently, it has been controversially discussed whether the defining motif for the fourth SCLC subtype is *YAP1*. *YAP1* expression is comparably low and has been predominantly observed in non–small cell components, as per immunohistochemistry analysis (4). Therefore, others have recently proposed to substitute the SCLC-Y subtype by either an SCLC-TN (“triple negative”) subtype or by an SCLC-I (“inflamed”) subtype, accounting for the absence of the three TFs *ASCL1*, *NEUROD1*, and *POU2F3* and the “inflamed” phenotype that is often observed in this fourth subtype (2, 5, 6). Recent studies, however, have highlighted that inflammatory characteristics are not exclusive to this fourth subtype but are also evident in other subtypes (6, 7). Also, SCLC-I would rather refer to active inflammation and immune infiltration as a characteristic of the tumor microenvironment. Therefore, compared with the SCLC-N, -A, and -P subtypes, the SCLC-I subtype is a tumor cell–extrinsic rather than tumor cell–intrinsic property. In contrast, the SCLC-Y subtype is a tumor cell–intrinsic subtype such as SCLC-N/A/P. For this study, we decided to adhere to the original four-way cancer cell–intrinsic NAPY subtype definition and investigate inflammatory phenomena in the tumor microenvironment, such as T-, B-cell inflammation and interferon (IFN)-I/II signaling as independent parameters.

Although the importance of these four TFs in SCLC has been widely demonstrated (8, 9) and links to cellular origins have been established (10), the characterization of downstream transcriptional programs linked to each of the TFs is less advanced and supported by only a few studies with limited patient numbers (11, 12). Moreover, a concrete standard protocol (including experimental process description and concrete computational procedures, including thresholds) for subtype assignments have not been defined. This potentially leads to insufficient comparability between studies and poor reproducibility. A standardized method for molecular classification of new SCLC samples is of utmost need, particularly considering the prognostic differences observed and the promising therapeutic vulnerabilities associated with these subtypes (5–7).

In this study, we used RNA sequencing (RNA-seq) gene expression data from a large cohort of real-world patients with SCLC to demonstrate the presence of downstream gene expression programs associated with the four key TFs: *NEUROD1*, *ASCL1*, *POU2F3*, and *YAP1*. We showed that these programs can distinguish the four molecular subtypes, largely congruent with the profiles of the four TFs. We built a robust machine learning classifier using these TF-associated downstream programs to demonstrate independent support for subtype assignment, complementing class assignment based solely on TF expression. We used our large real-word SCLC cohort to investigate a comprehensive collection of published signatures interrogating molecular or cellular phenomena related to cancer to reveal insights into the differential activity of molecular pathways across the four NAPY subtypes.

## Materials and Methods

This study used deidentified transcriptomic data (RNA-seq) from 460 SCLC samples from the Tempus database sequenced using the Tempus xR assay (Tempus AI, Inc.; Table 1). Briefly, Tempus xR is a whole-transcriptome RNA-seq assay (19,377 genes) utilizing formalin-fixed, paraffin-embedded (FFPE) tumor tissue. It achieves an average sequencing depth of 50 million reads per sample. The samples included in our analysis had a minimum of 15 million and a maximum of 80 million reads. Alignment to the human genome (GRCh37) is performed using STAR. Gene expression is quantified in transcript per million (TPM) using Kallisto, followed by log_2_ transformation with a pseudo-count of +1 (for details, see Supplementary Materials - RNA-seq Pipeline).

**Table 1.** Characteristics of patients in the Tempus SCLC cohort.

Characteristic	A.	B.	C.	D.
	*N* = 460^a^	*N* = 383^a^	*N* = 286^a^	*N* = 127^a^
Sex	​	​	​	​
Female	242 (53%)	199 (52%)	137 (48%)	65 (51%)
Male	218 (47%)	184 (48%)	149 (52%)	62 (49%)
Ethnicity	​	​	​	​
White	298 (87%)	247 (87%)	182 (85%)	84 (87%)
Black or African American	31 (9%)	27 (10%)	22 (10%)	9 (9%)
Other race	10 (3%)	7 (2%)	6 (3%)	2 (2%)
Asian	4 (1%)	4 (1%)	4 (2%)	2 (2%)
Unknown	117	98	72	30
Age (years)	​	​	​	​
60–69	203 (44%)	172 (45%)	129 (45%)	58 (45%)
70–79	118 (26%)	96 (25%)	68 (24%)	34 (27%)
50–59	91 (20%)	76 (20%)	58 (20%)	20 (16%)
≤49	26 (6%)	21 (6%)	19 (7%)	9 (7%)
80–89	20 (4%)	17 (4%)	11 (4%)	6 (5%)
Unknown	2	1	1	​
Smoking status	​	​	​	​
Current smoker/ex-smoker	163 (96%)	132 (96%)	93 (95%)	38 (97%)
Never smoker	7 (4%)	6 (4%)	5 (5%)	1 (3%)
Unknown	290	245	188	88
Lines of therapy completed	​	​	​	​
1	270 (63%)	204 (62%)	174 (61%)	122 (96%)
≥2	156 (37%)	124 (38%)	112 (39%)	5 (4%)
Unknown	34	55	​	​
Lines of therapy	​	​	​	​
Chemotherapy + ICI	158 (37%)	129 (39%)	127 (44%)	127 (100%)
Chemotherapy alone	133 (31%)	92 (28%)	65 (23%)	​
Different drug classes on different lots	131 (31%)	105 (32%)	93 (33%)	​
ICI alone	4 (1%)	2 (1%)	1 (0%)	​
Unknown	34	55	​	​
Disease stage	​	​	​	​
Stage IV	368 (87%)	308 (87%)	286 (100%)	127 (100%)
Stages I–III	53 (13%)	44 (13%)	​	​
Unknown	39	31	​	​
Disease recurrence	​	​	​	​
Primary	448 (100%)	373 (100%)	281 (100%)	124 (100%)
Unknown	12	10	5	3

A. Descriptive table of the complete Tempus SCLC cohort. B. Descriptive table of the Tempus SCLC subcohort with consensus NAPY subtypes used for downstream analyses comparing phenotype characteristics across subtypes. C. Descriptive table of the Tempus SCLC subcohort with consensus NAPY subtypes, restricted to stage IV patients with available clinical data for OS analysis, independent of therapy. D. Descriptive table of the Tempus SCLC subcohort with consensus NAPY subtypes, restricted to stage IV patients with available clinical data, treated exclusively with combinations of chemotherapy and ICIs, for a specific OS analysis targeting the most prevalent therapy scheme.

a *n* (%).

The SCLC subtype assignment for these samples was performed following the NAPY classification (*NEUROD1*-high, *ASCL1*-high, *POU2F3*-high, and *YAP1*-high; ref. 3), based on the highest, gene-wise z-scaled, log_2_-transformed TPM + 1 gene expression, and required the highest expression to be at least 0.3 *Z*-score units greater than the rest and not all-negative expressions. Samples that did not meet these requirements were not assigned to any subtype.

SCLC expression data were split at 80:20, with 20% spared out for the final classifier assessment. In 80% of patient records, in 20 cross-validation (CV) runs, linear support vector machine (SVM) classifiers were trained, excluding the four NAPY genes that had provided the class labels, with features selected and hyperparameters tuned in a nested CV (nCV) with outer fourfold CV executed on five random data splits and inner threefold CV (for details, see Supplementary Materials - ML Pipeline, Supplementary Figs. S1 and S2).

The classifiers utilized z-normalized gene expression of 80 predictor genes corresponding to four downstream programs and their 20 best marker genes, each associated with one of the four key TFs: *NEUROD1*, *ASCL1*, *POU2F3*, and *YAP1*. These programs were built based on the 20 genes with the strongest and exclusive association with one of the TFs, as determined by the Pearson correlation between the entire set of genes and the key TFs, in the training part of each of the 20 CV runs. This procedure yielded 20 slightly different groups of features × 4 × 20 genes.

Whereas the inner loop of our nCV served us to select the hyperparameters, the outer loop served us to estimate the accuracy of all the classifiers we built with the different group features. After model comparison, the final NAPY SVM classifier for application on further datasets was then generated using one of the groups of features that yielded the best-performing SVM classifiers during nCV and training on the full 80% of nCV data. External validation of the final NAPY SVM classifier was performed in the remaining 20% of the spared-out tumor samples that were not used for training.

We performed two additional external validation of the SVM classifier with 48 human SCLC cell lines from the Cancer Cell Line Encyclopedia (CCLE; ref. 13), publicly available on the Dependency Map (DepMap) Portal; and with 48 SCLC tumor samples from George and colleagues (14), publicly available from BioPortal. Gene expression TPM values of the protein-coding genes were inferred from RNA-seq data and reported after log_2_ transformation, using a pseudo-count of 1 as log_2_(TPM + 1) (13).

Subtype labels were obtained from the supplementary data of Rudin and colleagues (3). In their study, Rudin and colleagues assigned these labels based on the transcription regulator with the greatest relative overall expression—in the CCLE dataset, 26 SCLC-A, 11 SCLC-N, 4 SCLC-P, and 7 SCLC-Y, and in the George and colleagues dataset, 37 SCLC-A, 4 SCLC-N, 6 SCLC-P, and 1 SCLC-Y (see Supplementary Table S1).

Our curated RosettaSX compendium of cancer gene expression signatures (15), immune cell infiltration signatures (7, 16, 17), and a Notch signature (18) were evaluated in our Tempus SCLC cohort for differential expression across subtypes. Signatures were first filtered based on a coherence score higher than 0.2 to ensure their translatability in our dataset (15). For the signatures that passed this quality control, we estimated TF-associated pathway activity for each sample based on signature scores (for details, see Supplementary Materials - Cancer Pathway Activity Estimation - RosettaSX). Wilcoxon tests on the signature scores versus molecular subtypes were then performed in a pairwise manner to assess the significant expression variance across subtypes, with *P* values adjusted using the Bonferroni method. When multiple expression signatures represented the same biological process in the same context, a representative signature was selected based on the highest F value obtained by ANOVA of expression differences between subtypes. When multiple signatures were informed about the same upregulated or downregulated pathway, positively regulated pathways were selected to facilitate interpretation of the results.

Deidentified targeted DNA sequencing data for the same patients with SCLC in the Tempus database were analyzed. Tumors were profiled using the Tempus xT assay, which uses DNA isolated from FFPE tumor tissue to capture 598 or 648 genes, depending on assay version, with ∼500× coverage. We compared genomic alterations, including single-nucleotide variants and copy-number variations, across molecular subtypes. Fisher exact test was used to assess the association between gene mutations and molecular subtypes.

We analyzed overall survival (OS) in subgroups of our deidentified SCLC Tempus cohort using Kaplan–Meier analyses and Cox proportional hazards models as implemented in the coxph function from survival R package. OS times and events were defined by the time from the date of primary diagnosis or the biopsy collection date for molecular assessment (whichever was first) until the date of death. To determine OS, data on patient follow-up through medical records was used. Patients who were alive at the last follow-up were censored at that time point. Using coxph we estimate hazard ratios (HR) between subtypes, and analogously assess the effect of demographic covariates, as well as the influence of T-effector and tumor-associated macrophage (TAM) signals on survival, based on findings from studies with independent cohorts (7). We use the Wald test as an indicator for statistical significance of factors in coxph models.

This study was conducted on deidentified health information subject to an Institutional Review Board–exempt determination (Advarra Pro00072742) and did not involve human subject research.

## Results

### Classification of SCLC subtypes by TF-associated expression signatures

Using the expression profiles of the four NAPY TF genes, we determined the class labels for 332 of 460 SCLC samples based on our requirement for strong and selective expression of one of the four TFs. This procedure rigorously selects SCLC cases that can be assigned unambiguously to one of the four TFs to provide a clean basis for computational modeling and benchmarking in our study. The proportions of each molecular subtype among the 332 NAPY tumors in the Tempus SCLC dataset were 105 SCLC-N (32%), 111 SCLC-A (33%), 44 SCLC-P (13%), and 72 SCLC-Y (22%). This subtype distribution was consistent with other reported studies, showing a higher proportion of SCLC-N and SCLC-A subtypes and less than 40% of the cases for the SCLC-P and SCLC-Y subtypes (5). The 128 unlabeled cases represent a twilight zone in which cancers cannot unequivocally be attributed to a single TF subtype. They were set aside for an evaluation of how well our classification approaches could help in the diagnosis of SCLCs that are difficult to assign to a single NAPY class. Our 332 SCLCs with unambiguously assigned NAPY labels were used for the machine learning (ML) pipeline.

For the SVM classifier training and assessment, we split the 332 patient records in an 80:20 ratio. The smaller part of 20% of the data was left untouched and was not used in any feature selection and classifier training but was solely used for benchmarking of the final NAPY classifier. 80% of the data were used for a nCV approach for classifier training and assessment, and in each CV run, we trained SVMs for the prediction of NAPY labels for SCLCs, excluding the four NAPY genes that had provided the class labels. From the universe of genes, the feature selection was limited to 20 genes per class. For each NAPY class, a set of 20 selected genes can be considered a NAPY subtype-specific gene expression signature. Feature selection and hyperparameter tuning were performed in the inner CV runs, and classifier assessment in the outer CV runs, enabling unbiased classifier benchmarking. The final NAPY SVM classifier was obtained by training on the full training data (80%) using the 4 × 20 genes that yielded the best-performing SVM classifiers during nCV runs. The final NAPY SVM classifier was benchmarked on three datasets: on the 20% SCLC patient records saved for assessment, on the SCLC subsection of the CCLE dataset, and on the George and colleagues, 2015 tumor samples. The classification performance of the final NAPY SVM classifier derived from the nCV training on 80% of Tempus records and from the final SVM-assessment phase on 20% of Tempus spared-out records, and the performance results obtained from the additional validation on the CCLE cell lines and George and colleagues, tumors are presented in Table 2.

**Table 2.** Benchmarking our SCLC-NAPY ML classifier.

Metric	nCV training on 80% Tempus records	Assessment on 20% Tempus spared-out records	Further validation on CCLE cell lines	Further validation on George et al tumors
	(*n* = 264)	(*n* = 68)	(*n* = 48)	(*n* = 48)
Accuracy	0.87	0.90	0.92	0.79
Balanced accuracy	0.82	0.87	0.95	0.94
f_meas	0.84	0.89	0.88	0.72
Npv	0.96	0.97	0.97	0.88
Ppv	0.88	0.92	0.87	0.64
Precision	0.88	0.92	0.87	0.64
Recall	0.82	0.87	0.92	0.93
Sens	0.82	0.87	0.92	0.93
Spec	0.95	0.96	0.98	0.94

Classification performance results obtained with the SVM classifier trained on 80 genes (four downstream programs of 20 genes each). To obtain performance parameters, we used a nCV approach on training data and relied on assessment in independent data sets that were external to classifier training. The results were obtained by averaging the per-class performance metrics.

Final NAPY SVM classifier, cost = 0.031, features = 80 genes.

The models predicted the NAPY subtypes with a balanced accuracy of 82% and specificity of 95% on the nCV training data and 87% and 96% for the respective metrics on the independent dataset of 20% of spared-out records. These comparable performances between the nCV training phase and the assessment of the final NAPY classifier on left-out data provide evidence of little bias during classifier training. In addition, the balanced accuracy of 95% and specificity of 98% obtained from the evaluation in CCLE cell lines and the balanced accuracy and specificity of 94% in the George and colleagues, tumors demonstrated the strong predictive capability of the model using independent data.

**Figure 1.**
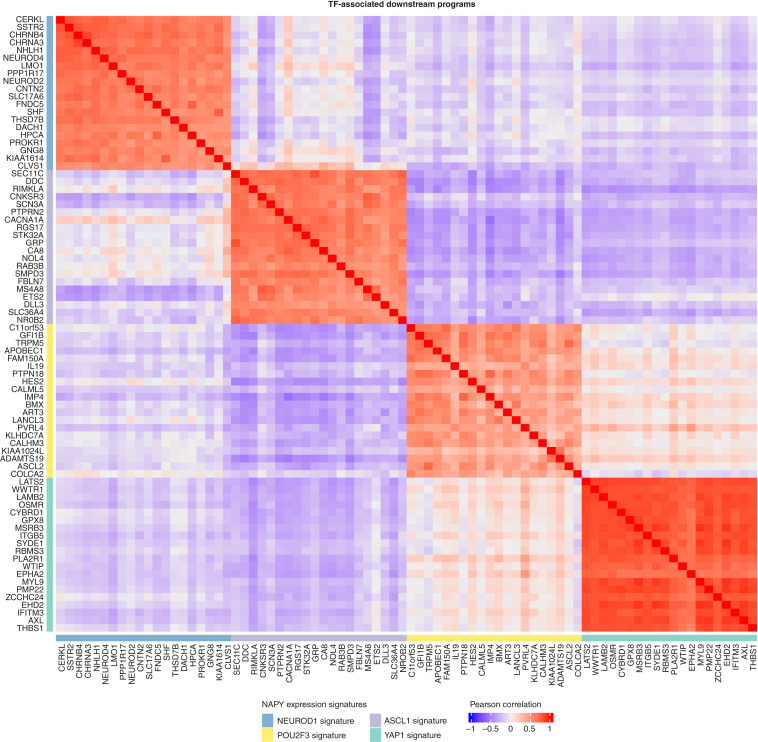
Correlation of NAPY signature genes across NAPY subtypes. Clustered heatmap of Pearson correlation coefficients between expression vectors of all possible gene pairs of the four TF-associated 20-gene downstream programs in the 80% Tempus nCV training data. We observe a clustering of genes by the NAPY subtype that a gene stands for, and strong signals in squares along the diagonal that mark the four coexpressed gene sets.

To visualize the signals of our four 20-gene transcriptional downstream programs leading to the favorable performance of the NAPY subtype classifier, we present a heatmap (see Supplementary Fig. S3) displaying the underlying expression patterns (*z*-scores) of the 4 × 20 genes across the 332 SCLCs used for training. Notably, strong signals appeared in four rectangles along the diagonal that originated from the selective expression of the four sets of genes in the four NAPY classes. In a clustered heatmap that shows the correlation coefficients between expression vectors of all possible gene pairs of the 4 × 20 genes, we observed a clustering of genes by the NAPY subtype, in which a gene stands for, and strong signals in squares along the diagonal that mark the four coexpressed gene sets (see Fig. 1).

The per-class performance results for the hold-out 20% of the Tempus records and for the CCLE and George and colleagues, datasets are presented in Supplementary Table S2. The classifier achieved per-class balanced accuracies ranging from 89% to 99% and per-class specificity results ranging from 73% to 100%, measured as one-vs-rest binary classification. Of the 68 samples from the reserved 20% of the Tempus records, 61 were correctly classified, whereas 44 of 48 samples from the CCLE dataset and 38 of 48 samples from the George and colleagues dataset were correctly classified. Detailed by-class results are available in the corresponding confusion matrices in Supplementary Tables S3–S5.

The precision (positive predictive value, PPV) for the SCLC-P subtype was low in the CCLE dataset, likely due to the imbalanced class distribution (26:11:4:7) and the small sample size for SCLC-P (only four samples). This small size can significantly affect performance, as even a single false positive from other groups can lower the PPV. We anticipate that a more balanced distribution with more SCLC-P samples in the validation set could improve the precision for SCLC-P classification.

Similarly, the low precision for SCLC-N, SCLC-P, and SCLC-Y and the low negative predictive value for SCLC-A in the George and colleagues dataset can be better understood by examining the confusion matrix (see Supplementary Table S5). The divergent classification of SCLC-A reduces the precision for all other subtypes due to the strong class imbalance in the original annotation for this dataset. Rudin and colleagues (2019) annotate a substantially higher frequency of SCLC-A (77%) compared with our NAPY classifier and less often call SCLC-N (8%), SCLC-P (13%), and SCLC-Y (2%) classes. There are various reasons which could explain the classification mismatch between our method and the method of Rudin and colleagues. These remain to be addressed in future studies, e.g., different vulnerabilities of the classification methods to class imbalances; tumor heterogeneity, especially in some of the SCLC-A tumors; or an absence of functional impact of high TF expression (here mostly for *ASCL1*) on the regulation of downstream gene sets.

The fact that performance scores from the application of our NAPY SVM model on Tempus patients with cancer, the CCLE cell lines, and the George and colleagues tumors are predominantly between 80% to 100% is a remarkable confirmation that our ML-based approach based on the four NAPY gene expression signatures as features can be translated between different datasets generated on different types of SCLC biospecimens. To further validate the biological relevance of the genes within our NAPY signatures, we benchmarked their overlap with known canonical targets of *NEUROD1*, *ASCL1*, *POU2F3*, and *YAP1*, as defined by chromatin immunoprecipitation sequencing (ChIP-seq) data (19). These results revealed high concordance with predicted target gene sets, with an overlap of 20 of 20 for *NEUROD1*, 19 of 20 for *ASCL1*, 15 of 20 for *POU2F3*, and 19 of 20 for *YAP1*. The four TF-associated downstream programs used as predictors in the SVM classifier are listed in Supplementary Table S6, along with several independent sources describing these genes in the context of SCLC (12, 20).

In addition, we predicted NAPY classes for the 128 excluded cases for which we were unable to unambiguously assign TF class labels based on stringent criteria. We compared the subtype predicted by our NAPY SVM classifier with the subtype determined using a more relaxed procedure compared with our first approach, which involved using the highest *z*-score expression among the TFs without any additional requirements. This comparison yielded a consensus classification in 58% of borderline cases (74 of 128). The subtypes obtained using these two methods, along with the expression patterns of our NAPY signatures, are provided in Supplementary Fig. S4. We used the *χ*^2^ test of independence to confirm that the results from these two methods based on either the TF expression or the expression of the downstream TF programs (excluding the TF) in our borderline cases were significantly associated (*P* = 1.67e−08), indicating that the observed frequencies of consensus subtypes were strongly enriched (see Supplementary Fig. S5). These findings suggest that the number of truly unclassifiable SCLC cases in our Tempus cohort may be reduced to *n* = 54. It also demonstrates how the parallel classification of NAPY subtypes by the analysis of TF expression and their downstream expression programs via our classifier can generate more confidence when labeling otherwise ambiguous SCLCs.

Based on these findings, we applied a consensus approach to our complete Tempus SCLC cohort (*n* = 460) by applying and comparing the class labels of both the TF-based and ML/signature-based methods. For 383 of 460 SCLCs, we obtained a consensus between the methods (see Supplementary Table S7). In the following, we utilized only these consensus samples for the subsequent analysis of patterns across NAPY subtypes. The distribution of consensus subtype samples (*n* = 383) was 150 SCLC-A (39%), 118 SCLC-N (31%), 36 SCLC-P (9%), and 79 SCLC-Y (21%).

### Assessing gene expression signatures for cancer signaling pathways and immune infiltration in NAPY subtypes

**Figure 2.**
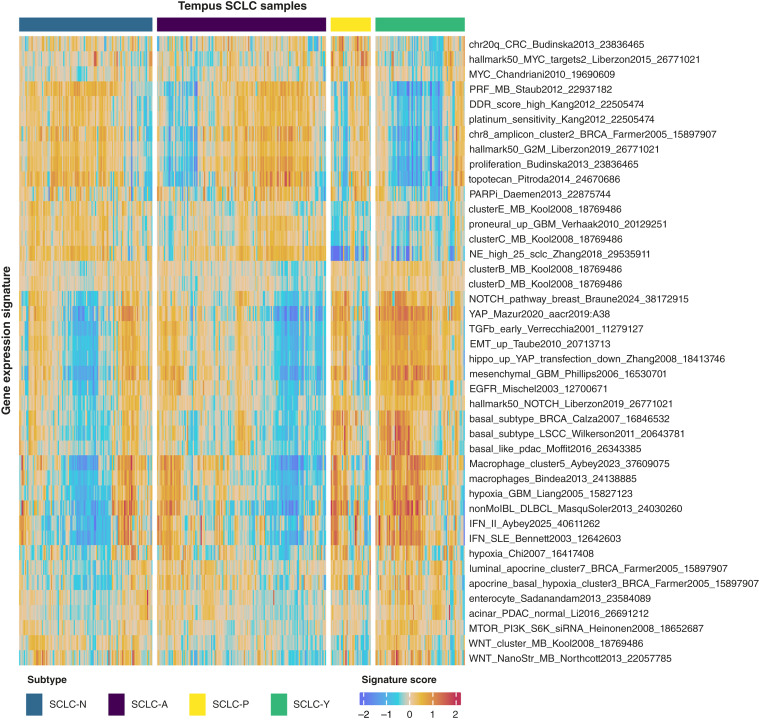
RosettaSX signatures in Tempus cohort across NAPY subtypes. Heatmap of per-sample signature expression across Tempus SCLC consensus NAPY subtypes (*n* = 383) for 43 relevant biological pathways. The per-sample signature score is calculated as the average *z*-score of the signature genes, for each sample. All signatures displayed in the heatmap demonstrated strong coherence scores (CS >0.2), indicating consistent expression changes within the signature gene set across samples, thereby underscoring the relevance of these signatures in our Tempus dataset. All signatures passed our filtering step of differential expression (*P* value < 0.05) in at least three of the six pairwise comparisons by Wilcoxon test.

We performed a RosettaSX gene expression signature analysis to explore the relationships between our four transcriptional subtypes and other strong and relevant gene expression programs in our set of SCLCs (see Fig. 2; Supplementary Figs. S6 and S7; ref. 15). Only signatures that showed significant differences in at least three of the six pairwise comparisons of subtypes, with a Bonferroni-adjusted *P* value lower than 0.05, were retained.

In accordance with previous studies, our findings demonstrate that cancers of the SCLC-N and SCLC-A subtypes exhibit a stronger neuroendocrine gene expression footprint than cancers of the SCLC-P and SCLC-Y subtypes, as detected here by significantly higher scores in typical signatures to indicate neural or neuroendocrine phenotypes, as measured by the neuroendocrine signature of Zhang and colleagues (21) and the proneural signature for glioblastoma by Verhaak and colleagues (22). We observed a similar association for signatures that typically indicate cell-cycle activity or cell proliferation in tissues, such as the medulloblastoma proliferation signature of Staub (23), proliferation signature from Budinska and colleagues (24), cell cycle G2M transition hallmark signature from Liberzon and colleagues (25), and DNA damage response pathway of Kang and colleagues (26). Higher cell proliferation activity is a known feature of neuroendocrine cancer types. In addition, we found chemosensitivity signatures such as platinum and topotecan sensitivity signatures from Kang and colleagues (26) and Pitroda and colleagues (27), respectively, highly correlated to these proliferation signatures and were predominantly expressed in SCLC-N and SCLC-A subtypes.

Conversely, several immune response signatures displayed the opposite behavior. As expected, the SCLC-Y subtype showed the strongest association with high immune/inflammatory signature scores. Evidence for a preference for high immune signatures with the Y-subtype is the significantly higher scores in the IFN signatures of Bennett and colleagues (28), Aybey and colleagues (17), and Staub (23), corroborating results from previous studies in which even the renaming of this subtype from SCLC-Y to SCLC-I has been proposed (5, 29). In addition, macrophage infiltration, as measured by our recently published macrophage cell type signature in Aybey and colleagues (16) and by Bindea and colleagues (30) signature, exhibited higher scores for the non-neuroendocrine phenotypes (SCLC-Y and SCLC.P). Similar findings for immune response–related genes have been previously described (5, 21, 29). However, the signals of inflammation and immune infiltration were not restricted to the SCLC-Y subtype. Other subtypes included cases with strong signs of immune cell activity. In particular, the frequencies of the 5% top scores for these five signatures among the four subtypes were as follows: for the IFN signature from Bennett and colleagues (28), the distribution of the top 5% scores was SCLC-Y (53%), SCLC-P (26%), SCLC-N (16%), and SCLC-A (5%); for the IFN-I signature from Aybey and colleagues (17), the distribution for top scores was as follows: SCLC-Y (42%), SCLC-P (32%), SCLC-N (16%), and SCLC-A (11%); whereas for Staub (23), the top scores were SCLC-Y (42%), SCLC-P (26%), SCLC-N (21%), and SCLC-A (11%). For the macrophage signature of Aybey and colleagues (16), the top 5% scores were distributed as follows: SCLC-Y (58%), SCLC-P (11%), SCLC-N (16%), and SCLC-A (16%), whereas for the macrophage signature of Bindea and colleagues (30), the highest scores were SCLC-Y (47%), SCLC-P (21%), SCLC-N (21%), and SCLC-A (11%). The fact that the association between the SCLC-Y subtype and the immune-high phenotype is significant but far from perfect supports our decision to focus on SCLC-Y as the fourth SCLC subtype and not on SCLC-I. We also observed an enrichment of hypoxia characteristics for the non-neuroendocrine phenotypes, indicated by significantly higher scores in the hypoxia signature for glioblastoma by Liang and colleagues (31) and Chi and colleagues (32).

Furthermore, our results demonstrated significantly higher scores for the SCLC-Y subtype in several signatures associated with distinct signaling pathway activities. The SCLCs of the SCLC-Y subtype exhibited strong signals of the epidermal growth factor receptor (*EGFR*) pathway signature of Mischel and colleagues (33), the Hippo pathway signature of Zhang and colleagues (21), and the PI3K signature of Heinonen and colleagues (34), which is in congruence with several previous reports (29, 35, 36). The activation of the PI3K–AKT–mTOR pathway has been implicated in proliferation and resistance to apoptosis in SCLC (1). The SCLC-Y subtype also showed the highest score among all subtypes for the YAP signature of Mazur and colleagues (37), which has been the result of YAP gene dependency in pooled CRISPR screen data, thereby providing independent functional credibility to our SCLC-Y classification. We observe a similar association with the SCLC-Y subtype for a group of signatures related to a mesenchymal character of tumor tissues: among these are signatures for transforming growth factor-β of Verrecchia and colleagues (38), and epithelial–mesenchymal transition (EMT) of Taube and colleagues (39). Whereas SCLC-Y tumors display the highest scores in these signatures, the SCLC-P subtype cancers often yield high signals. For the EMT signature, we find that SCLC-Y has the strongest mesenchymal expression characteristics. SCLC-A/-N tumors are rather epithelial-like, which is in concordance with other studies (5, 21, 29). We found that WNT pathway signature scores significantly higher for SCLC-N and SCLC-Y subtypes compared with the other subtypes, as evidenced by higher scores on the Kool and colleagues (40) and Northcott and colleagues (41) WNT pathway signatures on medulloblastoma. The WNT pathway has been reported in SCLC to play a role in chemoresistance acquisition (1). This chemoresistance has been reported to be associated with the non-neuroendocrine phenotypes and mesenchymal SCLC variants (5).

We found that the subtypes with strong neuroendocrine phenotypes (SCLC-A and SCLC-N) exhibited lower scores for the Notch signaling pathway signatures in both Braune and colleagues (18) and Liberzon and colleagues (25). These results are consistent with the fact that Notch signaling is a key negative regulator of neuroendocrine differentiation in SCLC (21). Our findings indicate that both MYC signatures of Liberzon and colleagues (25) and Chandriani and colleagues (42) are predominantly highly expressed in the SCLC-P subtype and correlate well with proliferation signatures. SCLC-P tumors are known to develop from a non-neuroendocrine chemosensory cell type called tuft cells (10). Some studies have already proposed a special role for the MYC pathway in SCLC-P tumors, which is supported by the results of our SCLC cohort (9, 10, 43).

The differentially expressed gene expression signatures identified in our Tempus cohort were further validated using two independent public datasets: CCLE SCLC cell lines and SCLC tumors from George and colleagues (2015) (Supplementary Figs. S8 and S9). Although most pairwise comparisons yielded nonsignificant *P* values due to the small sample sizes (limiting statistical power), the signatures showed similar trends across both the CCLE and George and colleagues datasets. Notably, expression patterns related to the EGFR, WNT, and NOTCH pathways observed as differentially expressed in our Tempus cohort were redetected in the George and colleagues cohort, a finding that could not be reproduced for the CCLE cell lines.

Our RosettaSX cancer pathway analyses revealed that multiple signatures were preferentially expressed in certain subtypes. However, most of them are not exclusively enriched in a single subtype, nor do they exhibit complete homogeneity across samples within that subtype. Although the imperfect matching of specific phenomena with NAPY subtypes could point to biological independence between preferred subtypes and phenomena, this observation could also be caused by differential degrees of tumor heterogeneity in SCLCs that have been described in other studies (4, 6). Evolutionary trajectories have been proposed as a potential cause of heterogeneity based on single-cell studies (9, 29). Nevertheless, our study shows that, despite the potential presence of heterogeneity, we can still predict NAPY subtypes with high accuracy from downstream gene expression patterns.

### Assessing frequencies of genomic alterations in NAPY subtypes

**Figure 3.**
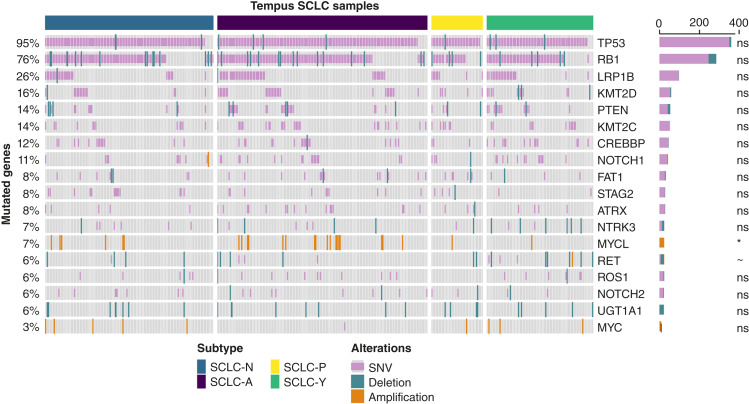
Oncoprint of genomic alterations across SCLC consensus NAPY subtypes in the Tempus cohort. We show the results for genomic alterations with a mutation frequency greater than 5%. Alterations in the MYC gene are included for informational purpose. Single-nucleotide variants (SNV) are in pink, copy-number deletions in blue, and copy-number amplifications in orange. We include *P* values from Fisher exact test to assess the potential association between gene-wise mutations and molecular subtypes, here included as row annotations (right side of plot), represented with the following symbols: ns, nonsignificant; *, *P* value < 0.05; ∼, 0.05 < *P* value < 0.1. Fisher exact test revealed nonsignificant association between the observed mutations and the molecular subtypes, except for a significant enrichment of MYCL amplifications in the SCLC-A subtype (17/148) compared with SCLC-N (6/118), SCLC-P (1/36), and SCLC-Y (1/75) (*P* value = 0.02). A trend toward a higher mutational burden of RET in the SCLC-Y subtype (10/75) compared with SCLC-A (7/148), SCLC-N (5/118), and SCLC-P (2/36) (*P* = 0.08) was also observed.

The frequencies of most genomic alterations found in the 383 SCLC tumor samples were consistent with findings from previous studies (Fig. 3), with nearly universal inactivation of *TP53* (95%) and *RB1* (74%) genes (14). We also identified *LRP1B* as mutated at a high frequency (27%). *LRP1B* is presumed to be a tumor-suppressor gene in SCLC and other cancer types, and its alteration has been proposed to play an important role in SCLC progression (44, 45). We found mutations in *KMT2D* and *KMT2C* in all SCLC subtypes at overall frequencies of 17% and 15%, respectively. Both genes are associated with chromatin remodeling pathways, and their alterations have been described in SCLC to contribute to the inactivation of their tumor-suppressive function (1, 45, 46). We observed alterations in *PTEN* (14%) and *CREBBP* (11%), which have been proposed to be associated with increased proliferation and cancer cell survival in the former and aberrant chromatin modification in the latter (1, 14).

We found mutations in the NOTCH gene family, specifically in the *NOTCH1* gene, in 10% of our cases, whereas mutations in the *NOTCH2* gene were observed at a slightly lower frequency of 6%. NOTCH family genes have been reported to have an inhibitory or blocking effect on the expression of neuroendocrine genes in SCLC cells (1, 14). We found that alterations in NOTCH genes were distributed across all NAPY subtypes. MYCL amplification was observed in 6% of our SCLC samples, primarily associated with the SCLC-A subtype, followed by SCLC-N. *MYCL* amplification is associated with cell-cycle progression and cell growth (13, 20). We found gene amplification of *MYC* in 3% of the cases, associated with SCLC-N, SCLC-Y, and SCLC-P, and absent in the SCLC-A subtype. Mutually exclusive patterns regarding amplification of MYC family genes (*MYC*, *MYCL*, and *MYCN*) have also been reported in other SCLC studies, showing subtype associations similar to our findings (1, 43). Interestingly, the higher expression of the MYC hallmark signature in the SCLC-P subtype cannot be traced back to the higher rates of MYC gene amplification in SCLC-P in our study. However, our SCLCs with *MYC* or *MYCL* amplifications showed higher scores for the MYC hallmark gene expression signature than the wild-type (Supplementary Fig. S10). Applying Fisher exact test suggests an absence of significant associations between most observed mutations and the NAPY subtypes, except for a significant enrichment of *MYCL* amplifications in the SCLC-A subtype (17/148) compared with SCLC-N (6/118), SCLC-P (1/36), and SCLC-Y (1/75) (*P* value = 0.02). There was also a trend toward a higher mutation frequency of *RET* in the SCLC-Y subtype (10/75) compared with SCLC-A (7/148), SCLC-N (5/118), and SCLC-P (2/36) (*P* = 0.08).

In summary, the investigation of DNA-level alterations across SCLC cases did not reveal exclusivity of individual alterations with NAPY subtypes. Thus, the NAPY expression subtypes can be considered a complementary classification scheme for SCLC alongside the most prevalent DNA-level alterations.

### Assessing OS in subgroups of our real-world evidence cohort

**Figure 4.**
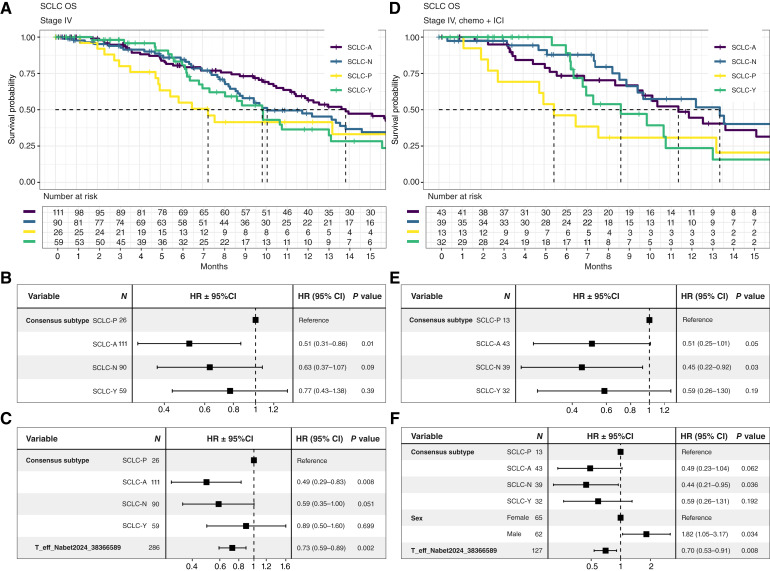
Survival analysis for NAPY subtypes in Tempus stage IV patients with SCLC. **A,** Kaplan–Meier estimates of OS across SCLC NAPY consensus subtypes: SCLC-A 13.8 (11.5, 17.2) months, SCLC-N 10.1 (8.8, 14.6) months, SCLC-P 7.3 (5.0, NA*) months, and SCLC-Y 9.9 (7.3, 15.6) months. **B,** Univariate Cox proportional hazards model results for evaluating the effect of molecular subtype on survival. HRs relative to SCLC-P, with 95% CI and statistical significance measured with Wald test *P* value. **C,** Multivariate Cox proportional hazards regression including T-effector score as covariate, based on its significant association found in the independent univariate analysis (Supplementary Table S8). **D–F,** Tempus SCLC patients with stage IV, restricted to chemo + ICI (*n* = 127) treatment. **D,** Kaplan–Meier estimates of OS across SCLC NAPY consensus subtypes: SCLC-A 11.4 (9.5, 16.8) months, SCLC-N 13.4 (9.5, 19.6) months, SCLC-P 5.5 (2.8, NA*) months, and SCLC-Y 8.7 (6.8, NA*) months. **E,** Univariate Cox proportional hazards model results for evaluating the effect of molecular subtype on survival. HRs relative to SCLC-P, with 95% CI and statistical significance measured with Wald test *P* value. **F,** Multivariate Cox proportional hazards regression including sex and T-effector score as covariates, based on their significant associations found in the independent univariate analysis (see also Supplementary Table S8). *, Insufficient number of events. CI, confidence interval.

We performed an analysis of OS in diverse subgroups of our Tempus SCLC cohort. We aimed to assess the utility of the NAPY subtypes and other biomarkers previously mentioned in the literature as prognostic biomarkers for SCLC. All OS analyses were focused only on stage IV patients, who comprise approximately 80% of our cohort, to avoid bias by including patients of different stages. We conducted analyses in two subcohorts: (C1) stage IV patients irrespective of treatment and (C2) stage IV patients restricted to patients receiving chemotherapy + immunotherapy checkpoint inhibitor (chemo + ICI) alone (*n* = 127). Only five of these 127 patients received multiple lines of chemo + ICI, but not chemo alone, and were therefore included as part this subgroup treated homogeneously with chemo + ICI; see Table 1 and Fig. 4. Patients that receive chemotherapy alone (*n* = 65) were not analyzed as an independent subgroup because the sample size is too small to yield meaningful results. By analyzing OS in cohorts C1 and C2, we aim to test whether individual associations remain stable when cohorts comprise patients who received different types of therapies versus cohorts with patients receiving only the most prevalent type of therapy, here chemo + ICI, thereby increasing the likelihood that findings can be attributed to intrinsic tumors traits, not variations in therapy across subgroups.

Using univariate and multivariate Cox regression models, we evaluated the association of molecular subtypes with OS. We performed pairwise comparisons of subgroups defined by NAPY classes. We identified poorer survival rates (HR <1) for the SCLC-P subtype compared with SCLC-A (HR = 0.51 with *P* = 0.01 for C1; HR = 0.51 with *P* = 0.05 for C2) and compared with SCLC-N subtype (HR = 0.63 with *P* = 0.09 for C1, HR = 0.45 with *P* = 0.03 for C2); see Fig. 4. Similar clinical outcomes, suggesting a worse prognosis for the SCLC-P subtype, have also been previously reported (5, 6). Separate univariate Cox regressions revealed that a low T-effector cell score (in C1) and a low T-effector cell score and male sex (in C2) as statistically significant markers for poor prognosis (OS), whereas the analysis of age and TAM scores did not show differential OS; see Supplementary Table S8. An association between T-effector cell gene expression signature scores and survival has been previously reported in an independent dataset (7). The observed prognostic effects persisted in Cox model analyses that comprise the number of lines of therapy (=1 vs. >1; all chemo + ICI) as an independent factor, which is not surprising as only 5 of the 127 patients received multiple lines of chemo + ICI.

Finally, we investigated SCLC-P (yes/no), sex (male/female), and T-effector cell expression signature scores in multivariate models for C1 and C2. Notably, SCLC-P, male sex, and low T-effector cell gene expression score remained significant as individual factors, suggestions that they act as independent poor prognosis biomarkers in SCLC; see Fig. 4.

## Discussion

In this study, we analyze one of the largest real-world SCLC cohorts for which gene expression and genomic alteration data are available. We developed four expression signatures associated with the four key TFs, *NEUROD1*, *ASCL1*, *POU2F3*, and *YAP1* (NAPY), that were instrumental in differentiating each of the four SCLC NAPY subtypes. They can be the basis for ML-based class prediction in gene expression data obtained from different biospecimens, as demonstrated here for complex tumor tissues sampled by SCLC biopsies and for cancer cell lines cultured under controlled conditions.

Each of our NAPY subtype–specific signatures comprises 20 genes, most of which have been previously identified as differentially expressed in a specific NAPY subtype by other research groups or overlap with known targets of the NAPY TFs based on complementary ChIP-seq data. Therefore, we are confident that these 4 × 20 genes constitute transcriptional programs that are selectively activated in the four different NAPY subtypes. The selection of *n* = 20 genes per class is motivated by the optimal performance of our classification approach at the lowest number of genes. We do not claim that the number of differentially regulated genes between SCLCs of different NAPY classes is limited to 20 genes only. Likewise, we also achieve only marginally worse prediction performance with *n* = 10 genes. Our preliminary assessment suggests that there are considerably more genes specifically expressed per class.

Some differentially expressed genes are not even intrinsically expressed in cancer cells, which is consistent with our observation of preferential (but not perfectly associated) immune gene signature expression in the Y-subtype. However, we are confident that our selection of the top 20 genes per class comprises predominantly intrinsically expressed marker genes, as the performance assessment of our ML model across independent datasets suggests that they can be used to infer NAPY classes in SCLC gene expression data obtained not only from patient biopsies but also from SCLC cell lines that are pure representations of cancer cells. Although we cannot claim that the 4 × 20 genes play a causative role in SCLC development, they can be regarded as important integrators of lineage-defining SCLC TF function, thereby representing entry points for further research on how phenotypic differences between NAPY subtypes emerge.

We believe that our ML approach for the prediction of gene expression program activity downstream of NAPY TFs extends the method space to diagnose SCLC more precise, offering complementary information compared with approaches relying solely on the gene or protein expression of the four TFs or on unsupervised clustering techniques based on highly variable genes. We therefore herein propose assigning molecular subtypes based on the consensus of predicted classes from both the TF-based and ML/signature-based methods. This approach enhances the accuracy of subtype assignment, facilitating the comparison and identification of phenotype characteristics of the NAPY classes. Examples of this improvement are illustrated in Supplementary Figs. S11 and S12, in which removing complex heterogeneous samples increases the capability to detect differential signals in parameters like OS and MYC-related gene signature activity among the SCLC groups.

Although we have demonstrated how the interrogation of our NAPY subtype-specific downstream programs can lead to complementary evidence of SCLC classification, we do not rule out the possibility of achieving further classification improvement with the inclusion of additional features obtained from technologies that interrogate other levels of biology, such as next-generation sequencing–based chromatin or methylation profiling, as these data modalities are also well suited for revealing cell-of-origin patterns in complex cancer tissues.

Furthermore, our four 20-gene NAPY signatures extend frameworks for the comprehensive characterization of gene expression through the analysis of published signature compendia, here our RosettaSX framework. Investigating published signatures across our NAPY classes using RosettaSX revealed differential scores for various cancer phenomena or pathways, such as WNT, MYC, EMT, EGF, NOTCH, neuroendocrine/proneural likeness, and immune cell infiltration, thereby providing a comprehensive picture of the cancer pathway activity across the four main SCLC subtypes.

Finally, our analysis identifies the SCLC-P subtype and low T-effector cell gene expression scores as independent biomarkers of poor prognosis in SCLC. We have shown this for patients with stage IV SCLC, independent of therapy, and for stage IV patients treated with combinations of chemotherapy and immunotherapy. These prognostic biomarkers and the multivariate prognostic model that integrates them warrant further evaluation in larger clinical studies, then comprising more patients with SCLC of lower stage and patients receiving different chemotherapies alone.

## Supplementary Material

Supplementary Data This file comprises the Supplementary Materials excluding Figures and Tables

Supplementary Figure S1 Cross-validation workflow.

Supplementary Figure S2 Feature Selection.

Supplementary Figure S3 Expression heatmap for 4x20 NAPY classifier genes in TEMPUS cohort.

Supplementary Figure S4 Heatmap of NAPY signature gene expression for borderline TEMPUS cases.

Supplementary Figure S5 Max-TF-based vs Signature-ML-based NAPY classification for hard TEMPUS cases.

Supplementary Figure S6 Selected signatures scored across NAPY subtypes in TEMPUS cohort.

Supplementary Figure S7 RosettaSX heatmap for TEMPUS SCLC consensus cohort.

Supplementary Figure S8 Selected signatures scored across NAPY subtypes in CCLE SCLC cell lines.

Supplementary Figure S9 Selected signatures scored across NAPY subtypes in George cohort.

Supplementary Figure S10 MYC expression signature versus MYC amplification status.

Supplementary Figure S11 Survival characteristics of SCLC NAPY subtypes

Supplementary Figure S12 MYC hallmark expression signature across NAPY subtypes

Supplementary Table S1 NAPY labels for independent validation.

Supplementary Table S2 Per-class classification performance on external data

Supplementary Table S3 Confusion matrix TEMPUS hold-out data.

Supplementary Table S4 Confusion matrix CCLE cell lines.

Supplementary Table S5 Confusion matrix George cohort.

Supplementary Table S6 Literature evidence of TF regulation for the four 20-gene NAPY gene expression signatures.

Supplementary Table S7 Confusion matrix TF-based vs ML-based NAPY classification.

Supplementary Table S8 Univariate survival analysis in Stage IV TEMPUS patients.

## Data Availability

Gene expression data used for independent validation of the SVM classifier is publicly available through cBioPortal (George and colleagues; ref. 14) and DepMapPortal (CCLE cell lines; ref. 13). Derived data supporting the conclusions of this article are included within the article and its additional files. Requests for access to further data should be sent to eike.staub@merckgroup.com. Restrictions, however, may apply to the availability of the molecular and clinical deidentified, individual-level SCLC data from Tempus AI, Inc. due to privacy and contractual reasons. The trained model, along with original code for its usage and reproduce the validation on the publicly available datasets, has been deposited on GitHub (https://github.com/nkiedanski/SCLC-Subtyping-publication.git).
