# Supplementary material for: Subclassification of Small Cell Lung Cancer Based on Gene Expression Signatures and Machine Learning
Source: Cancer Res Commun. 2026 Mar 12;6(3):545–56. doi: 10.1158/2767-9764.CRC-25-0512 (PMC13012008; doi:10.1158/2767-9764.CRC-25-0512)
Supplement: Supplementary Data — This file comprises the Supplementary Materials excluding Figures and Tables [file crc-25-0512_supplementary_data_suppsm.docx]

## Supplementary Materials

### RNA-Seq Pipeline

The data provider, Tempus AI, Inc., is a CLIA-certified and CAP-accredited laboratory. As such, its methods and laboratory protocols are developed, validated, and performed to meet federal standards for clinical testing and rigorous quality standards, ensuring the quality and reliability of its results.

The samples included in our analysis had a read depth ranging from a minimum of 15 million to a maximum of 80 million reads. Additionally, all included samples met the quality criterion of a median log-fold change distribution within ±0.3 of the overall median after three iterations, each utilizing a different random reference sample.

More details on Tempus internal assays have been previously published (Beaubier *et al*., 2019) (Leibowitz *et al*., 2022) (Michuda *et al*., 2023) (Wenric *et al*., 2023) (Zander *et al*., 2025) (Gai *et al*., 2025). Validation summary can be found in Tempus website.

### Machine Learning Pipeline

A nested cross-validation (nCV) approach was implemented to select the best model from several choices and robustly estimate its generalization error. The nCV approach enables both the optimization of hyperparameters and model selection without resulting in an overly optimistic generalization error due to overfitting, which can occur with a simple cross-validation approach (Cawley *et al.,* 2010). Briefly, the process of nCV involves nesting two cross-validation procedures: an outer cross-validation loop where the model is evaluated and an inner cross-validation loop from each training subset to evaluate hyperparameters and select the best hyperparameter for that run.

Our setting consisted of a 4-fold cross-validation in the outer loop, with 3-folds used for training and the remaining fold for testing. This process was repeated five times, resulting in 20 different outer cross-validation runs. In the inner loop, we used a 3-fold cross-validation, with 2-folds for training and the remaining fold for testing. This setup was based on 80% of our Tempus SCLC cohort, with the remaining 20% being spared out to evaluate the final model (see **Supplementary Figure S1**).

The predictor genes for the model were chosen from the universe of genes to ensure the representation of downstream programs associated with each of the four transcription factors: *NEUROD1, ASCL1, POU2F3* and *YAP1.* To achieve this, the Pearson correlation between all genes and each of the key transcription factors was measured in the training part of each of the 20 distinct configurations. The top genes were selected based on a strong positive and exclusive correlation with one of the four key transcription factors, thus defining the four TF-associated downstream programs (see **Supplementary Figure S2**). TFs were excluded from the model.

Gene expression was mean-centered and standardized (z-normalized) as a pre-processing step before training and running the models.

Different downstream program lengths, including 10-gene, 20-gene, 30-gene and 50-gene signatures, were assessed. The average f1 performance score across runs was found to be slightly inferior (83%) when 4x10 features were used to feed the SVM models (i.e. four 10-gene downstream programs). Average f1 performance score was 84% for SVM models using 80, 120, and 200 features (i.e. four downstream programs, each a 20-gene, 30-gene, 50-gene signature, respectively). Therefore, we concluded that incorporating larger gene sets did not further improve the classification performance, leading us to the selection of the simpler model with 80 features (four 20-gene downstream programs of 20 genes each).

The final NAPY SVM classifier for application on further datasets was generated using one of the groups of features of 4 × 20 genes that yielded the best-performing SVM classifiers during nested CV, and training on the full 80% of nested CV data.

The final NAPY SVM classifier was then evaluated in the remaining 20% of the Tempus spared-out samples that were not used for training, and in the two independent validation datasets.

We used R v4.4.0 and implemented the model using the tidymodels framework v1.1.0, kernlab v0.9-32, and rstatix v0.7.2.

### Cancer Pathway Activity Estimation - RosettaSX

Activity of gene expression modules of pathways for each sample was estimated based on signature scores. First, each gene was normalized using z-score normalization, scaling by its mean and standard deviation. Next, we averaged the normalized gene expression levels within each gene set to obtain a sample signature score. For each pathway, we then statistically compared signature scores across SCLC molecular subtypes using Wilcoxon test. While we acknowledge that alternative methods, such as GSVA or ssGSEA could also have been applied, we opted for this approach due to its simplicity, robustness, comparable performance and prior application in the RosettaSX signature analysis framework ([Kreis *et al.*, 2021](https://pubmed.ncbi.nlm.nih.gov/?term=Kreis+J&cauthor_id=34583245)).
