# Supplementary material for: Subclassification of Small Cell Lung Cancer Based on Gene Expression Signatures and Machine Learning
Source: Cancer Res Commun. 2026 Mar 12;6(3):545–56. doi: 10.1158/2767-9764.CRC-25-0512 (PMC13012008; doi:10.1158/2767-9764.CRC-25-0512)
Supplement: Supplementary Figure S1 — Cross-validation workflow. [file crc-25-0512_supplementary_figure_s1_suppsf1.pdf]

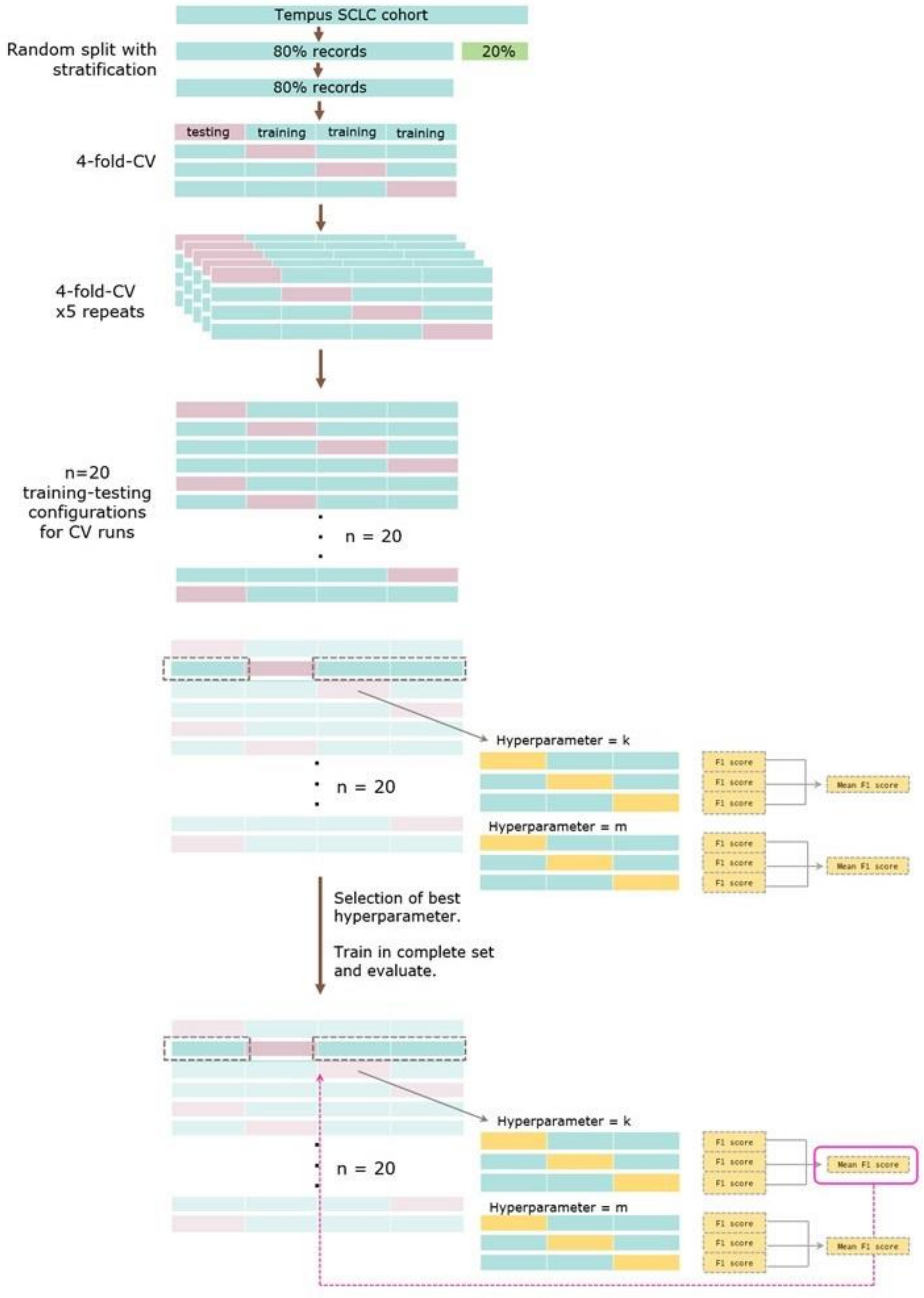

Supplementary Figure S1. Cross-validation workflow. Nested cross-validation setting developed on 80% of Tempus records, consisting of 4-fold CV per 5 repeats in the outer loop (20 outer loop CV iterations) for feature selection and performance evaluation, and 3-fold CV in the inner loop for hyperparameter optimization.
