# Supplementary material for: Subclassification of Small Cell Lung Cancer Based on Gene Expression Signatures and Machine Learning
Source: Cancer Res Commun. 2026 Mar 12;6(3):545–56. doi: 10.1158/2767-9764.CRC-25-0512 (PMC13012008; doi:10.1158/2767-9764.CRC-25-0512)
Supplement: Supplementary Figure S2 — Feature Selection. [file crc-25-0512_supplementary_figure_s2_suppsf2.pdf]

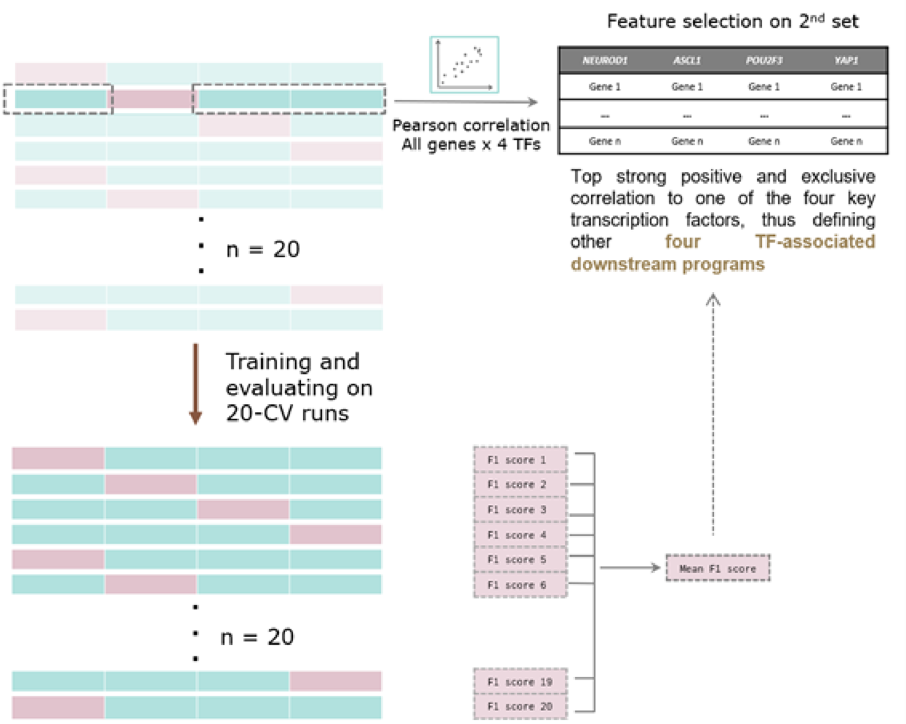

Supplementary Figure S2. Feature selection. Top genes were selected based on a strong positive, and exclusive association with one of the TFs, measured by Pearson correlation in the training data (k-1 folds) of each of the 20 CV configurations.
