# Supplementary material for: Subclassification of Small Cell Lung Cancer Based on Gene Expression Signatures and Machine Learning
Source: Cancer Res Commun. 2026 Mar 12;6(3):545–56. doi: 10.1158/2767-9764.CRC-25-0512 (PMC13012008; doi:10.1158/2767-9764.CRC-25-0512)
Supplement: Supplementary Figure S3 — Expression heatmap for 4x20 NAPY classifier genes in TEMPUS cohort. [file crc-25-0512_supplementary_figure_s3_suppsf3.pdf]

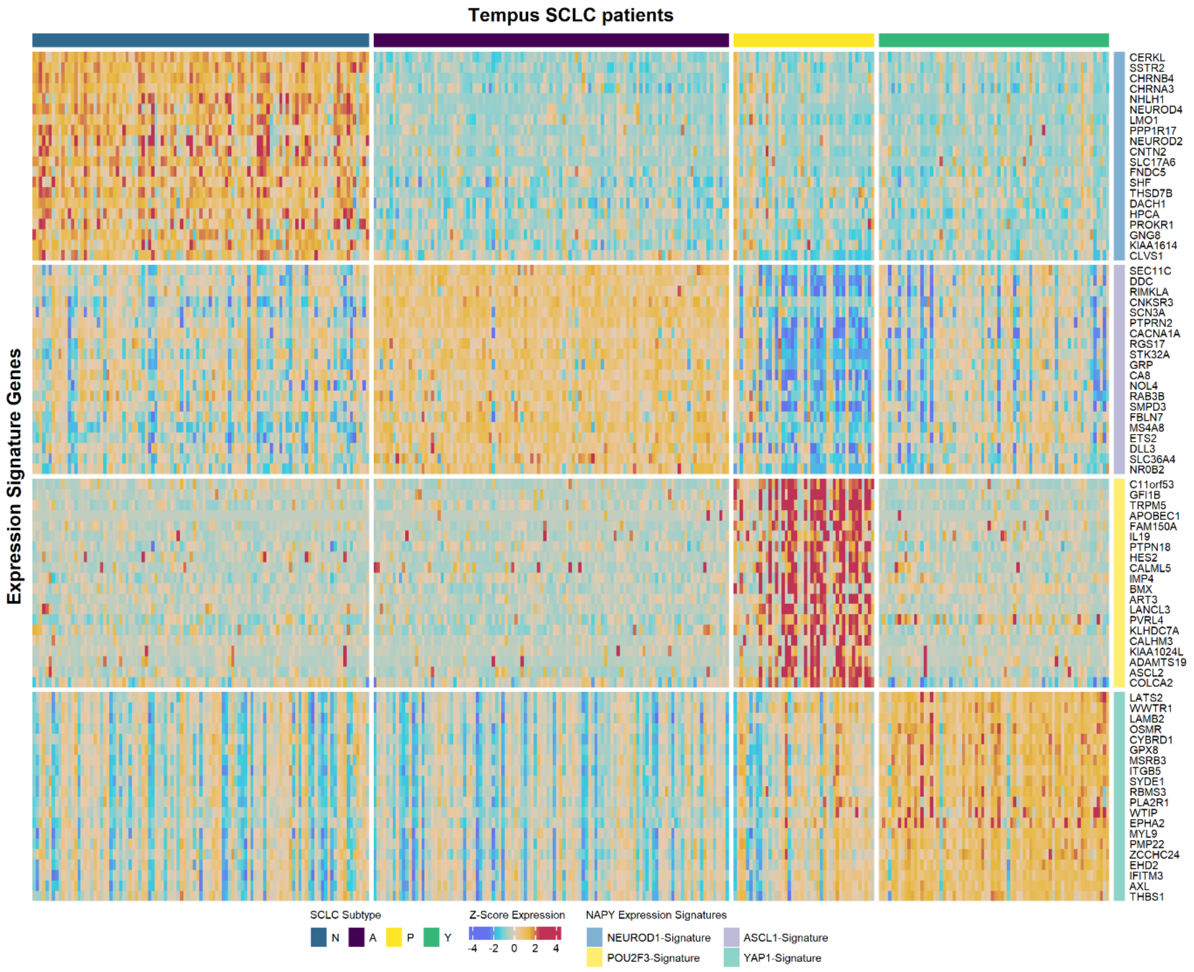

Supplementary Figure S3. Expression heatmap for 4x20 NAPY classifier genes in TEMPUS cohort. Heatmap of z-score expression for the 4x20 genes of our NAPY Signatures along the 332 Tempus SCLCs used for nCV training. Notably, strong signals appear in four squares along the diagonal, that originate from selective expression of the genes in the four NAPY classes.
