# Supplementary material for: Subclassification of Small Cell Lung Cancer Based on Gene Expression Signatures and Machine Learning
Source: Cancer Res Commun. 2026 Mar 12;6(3):545–56. doi: 10.1158/2767-9764.CRC-25-0512 (PMC13012008; doi:10.1158/2767-9764.CRC-25-0512)
Supplement: Supplementary Figure S4 — Heatmap of NAPY signature gene expression for borderline TEMPUS cases. [file crc-25-0512_supplementary_figure_s4_suppsf4.pdf]

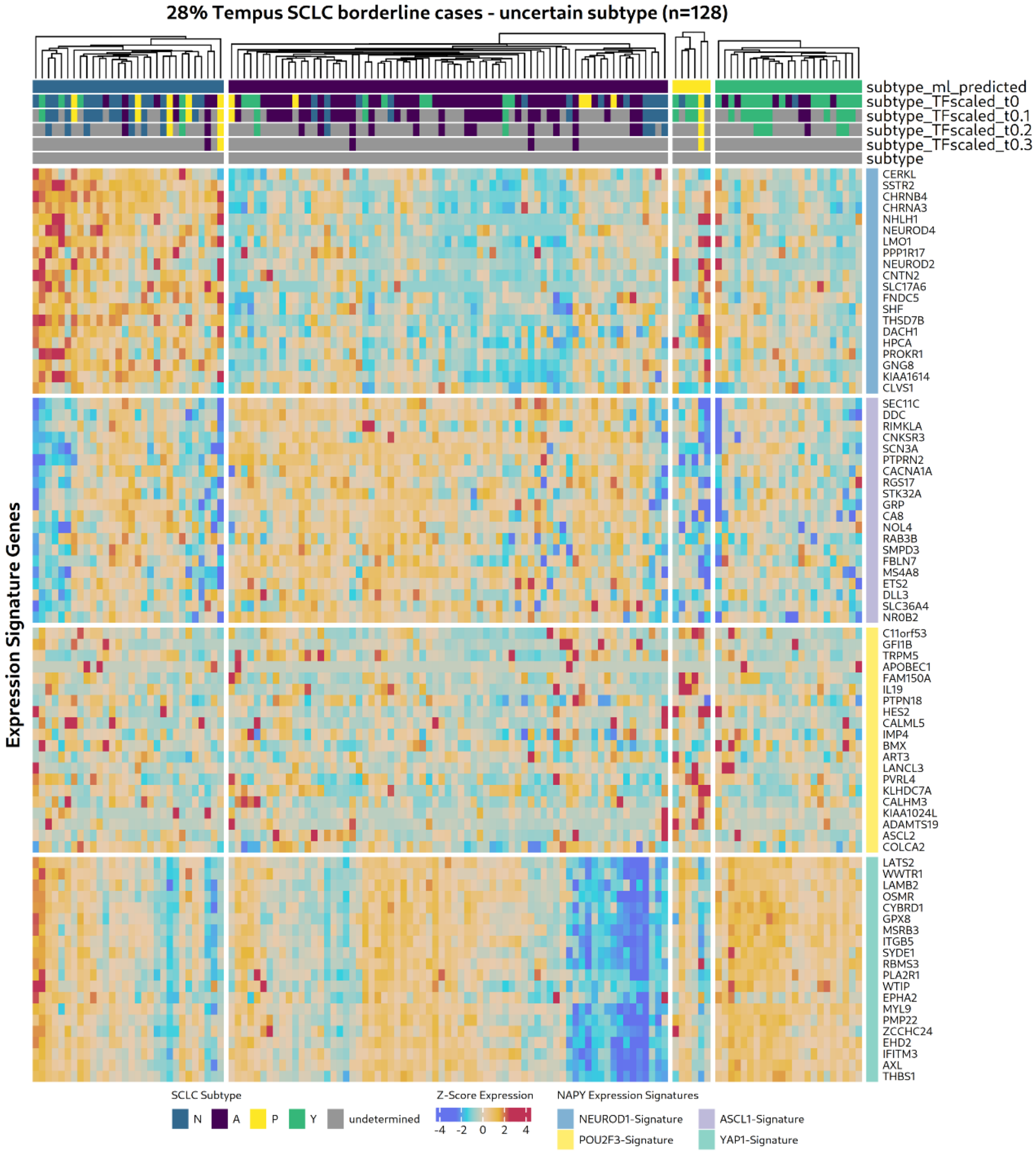

Supplementary Figure S4. Heatmap of NAPY signature gene expression for borderline TEMPUS cases. Heatmap showing the z-score expression of the 80 genes within our four subtype- associated gene expression signatures for the 28% Tempus SCLC borderline cases (n=128). Sample annotations consist of (from top to bottom): the subtype predicted by our NAPY SVM classifier; the subtype obtained based on the highest z-score TF-expression; the subtype obtained using the highest z-score TF-expression having a minimum absolute difference (threshold) varying from 0.1 to 0.3; and the subtype obtained using our established rule resulting in all 128 undetermined cases.
