# Supplementary material for: Subclassification of Small Cell Lung Cancer Based on Gene Expression Signatures and Machine Learning
Source: Cancer Res Commun. 2026 Mar 12;6(3):545–56. doi: 10.1158/2767-9764.CRC-25-0512 (PMC13012008; doi:10.1158/2767-9764.CRC-25-0512)
Supplement: Supplementary Figure S5 — Max-TF-based vs Signature-ML-based NAPY classification for hard TEMPUS cases. [file crc-25-0512_supplementary_figure_s5_suppsf5.pdf]

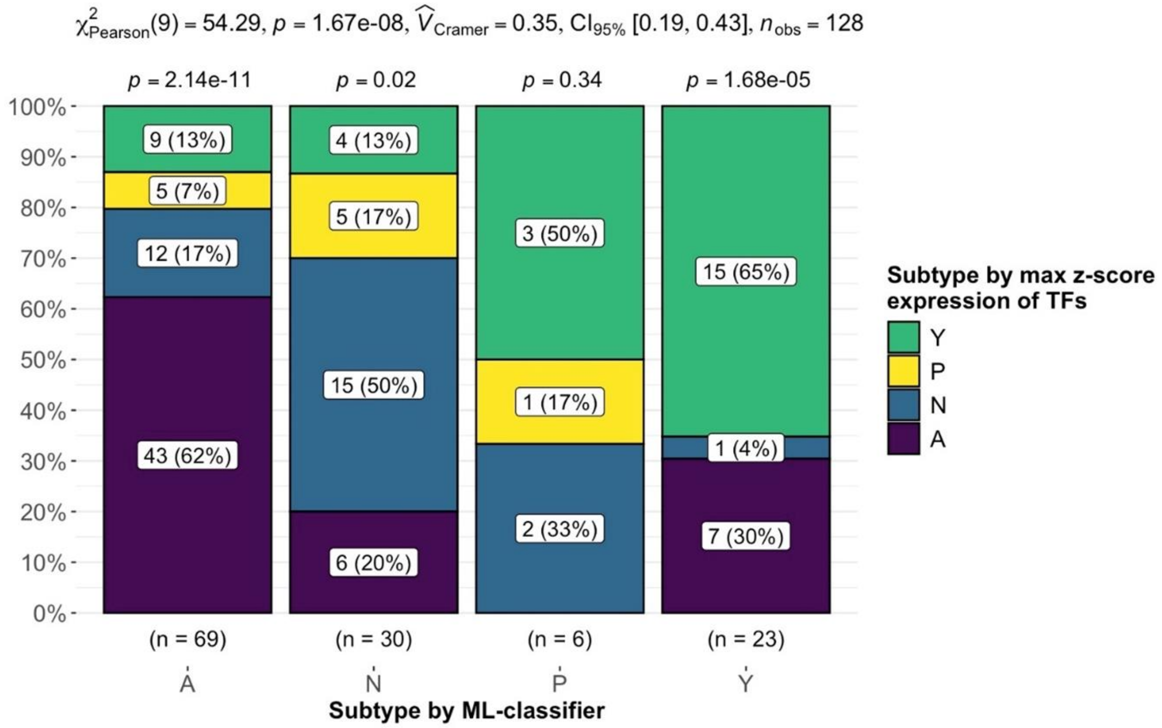

Supplementary Figure S5. Max-TF-based vs Signature-ML-based NAPY classification for hard TEMPUS cases. The bars on the x-axis represent the frequencies of subtypes as classified by our NAPY SVM classifier (SCLC-A: 69, SCLC-N: 30, SCLC-P: 6, SCLC-Y: 23). The colors of the bars (row direction) indicate the frequencies of subtypes observed using the highest z-score expression among transcription factors (TFs) without additional requirements (SCLC-A: 56, SCLC-N: 30, SCLC-P: 11, SCLC-Y: 31). Given the subtype obtained by the NAPY SVM classifier in the x-axis, each bar is subdivided on the y-axis to show the counts and percentages of subtypes observed using the highest z-score expression among the TFs, thus illustration the contingency table for the 128 borderline cases in our Tempus SCLC cohort. A Chi-square test of independence was conducted between the results of the two methods, confirming their association ($\chi 2=54.29$, p-value=1.67e-08). The Cramer's V statistic, with a 95% confidence interval, indicates a moderate strength of association ($\hat{V} = 0.35$) between the variables. The p-values displayed above the bars indicate whether there are significant differences in the proportions of subtypes within each group. Low p-values (< 0.05) suggest that the distribution of subtypes in that group significantly differs from what would be expected under the null hypothesis of no relationship. Note that the TF-based predictions most often match the best TF-based predictions even when the best and second-best TF-based class assignments are close, and the class assignment based on TF expression alone can be regarded as ambiguous. Figure generated with ggstatsplot::ggbarstats() from Patil *et al.,* 2021.
