# Supplementary material for: Subclassification of Small Cell Lung Cancer Based on Gene Expression Signatures and Machine Learning
Source: Cancer Res Commun. 2026 Mar 12;6(3):545–56. doi: 10.1158/2767-9764.CRC-25-0512 (PMC13012008; doi:10.1158/2767-9764.CRC-25-0512)
Supplement: Supplementary Figure S7 — RosettaSX heatmap for TEMPUS SCLC consensus cohort. [file crc-25-0512_supplementary_figure_s7_suppsf7.pdf]

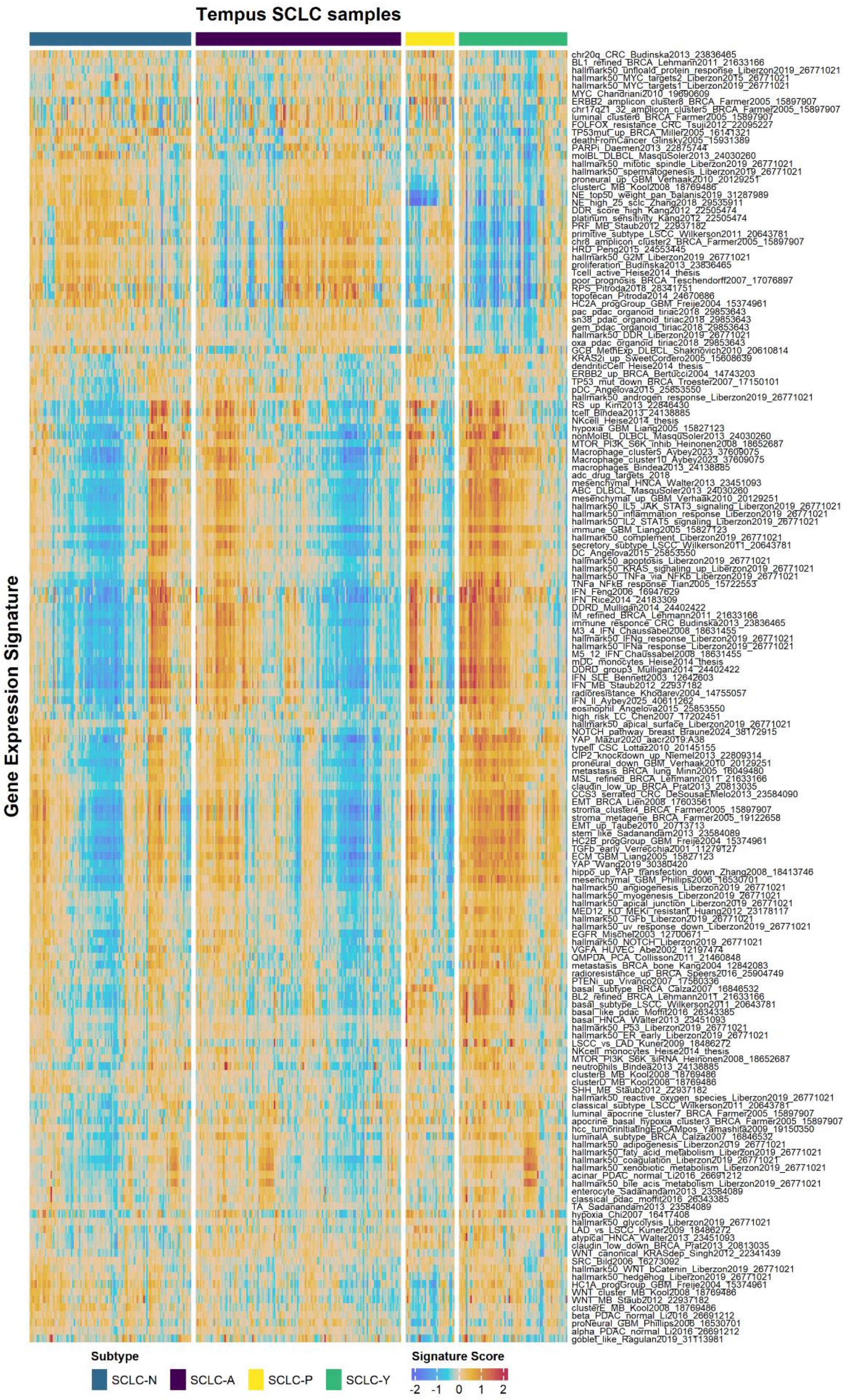
Tempus SCLC samples
Gene Expression Signature
chr20q_CRC_Budinska2013_23836465
BL1_refined_BRCA_Lehmann2011_21633166
hallmark50_unfloald_protein_response_Liberzon2019_26771021
hallmark50_MYC_targets2_Liberzon2015_26771021
hallmark50_MYC_targets1_Liberzon2019_26771021
MYC_Chandriani2010_19690609
ERBB2_amplicon_cluster8_BRCA_Farmer2005_15897907
chr17q21_32_amplicon_cluster5_BRCA_Farmer2005_15897907
luminal_cluster6_BRCA_Farmer2005_15897907
FOLFOX_resistance_CRC_Tsuji2012_22095227
TP53mut_up_BRCA_Miller2005_16141321
deathFromCancer_Glinsky2005_15931389
PARPi_Daemen2013_22875744
molBL_DLBCL_MasquSoler2013_24030260
hallmark50_mitotic_spindle_Liberzon2019_26771021
hallmark50_spermatogenesis_Liberzon2019_26771021
proneural_up_GBM_Verhaak2010_20129251
clusterC_MB_Kool2008_18769486
NE_top50_weight_pan_balanis2019_31287989
NE_high_25_sclc_Zhang2018_29535911
DDR_score_high_Kang2012_22505474
platinum_sensitivity_Kang2012_22505474
PRF_MB_Staub2012_22937182
primitive_subtype_LSCC_Wilkerson2011_20643781
chr8_amplicon_cluster2_BRCA_Farmer2005_15897907
HRD_Peng2015_24553445
hallmark50_G2M_Liberzon2019_26771021
proliferation_Budinska2013_23836465
Tcell_active_Heise2014_thesis
poor_prognosis_BRCA_Teschendorff2007_17076897
RPS_Pitroda2018_28341751
topotecan_Pitroda2014_24670686
HC2A_progGroup_GBM_Freije2004_15374961
pac_pdac_organoid_tiriac2018_29853643
sn38_pdac_organoid_tiriac2018_29853643
gem_pdac_organoid_tiriac2018_29853643
hallmark50_DDR_Liberzon2019_26771021
oxa_pdac_organoid_tiriac2018_29853643
GCB_MethExp_DLBCL_Shaknovich2010_20610814
KRAS2i_up_SweetCordero2005_15608639
dendriticCell_Heise2014_thesis
ERBB2_up_BRCA_Bertucci2004_14743203
TP53_mut_down_BRCA_Troester2007_17150101
pDC_Angelova2015_25853550
hallmark50_androgen_response_Liberzon2019_26771021
RS_up_Kim2013_22846430
tcell_Bindea2013_24138885
NKcell_Heise2014_thesis
hypoxia_GBM_Liang2005_15827123
nonMolBL_DLBCL_MasquSoler2013_24030260
MTOR_PI3K_S6K_inhib_Heinonen2008_18652687
Macrophage_cluster5_Aybey2023_37609075
Macrophage_cluster10_Aybey2023_37609075
macrophages_Bindea2013_24138885
adc_drug_targets_2018
mesenchymal_HNCA_Walter2013_23451093
ABC_DLBCL_MasquSoler2013_24030260
mesenchymal_up_GBM_Verhaak2010_20129251
hallmark50_IL5_JAK_STAT3_signaling_Liberzon2019_26771021
hallmark50_inflammation_response_Liberzon2019_26771021
hallmark50_IL2_STAT5_signaling_Liberzon2019_26771021
immune_GBM_Liang2005_15827123
hallmark50_complement_Liberzon2019_26771021
secretory_subtype_LSCC_Wilkerson2011_20643781
DC_Angelova2015_25853550
hallmark50_apoptosis_Liberzon2019_26771021
hallmark50_KRAS_signaling_up_Liberzon2019_26771021
hallmark50_TNFa_via_NFKb_Liberzon2019_26771021
TNFa_NFkB_response_Tian2005_15722553
IFN_Feng2006_16947629
IFN_Rice2014_24183309
DDRD_Mulligan2014_24402422
IM_refined_BRCA_Lehmann2011_21633166
immune_responce_CRC_Budinska2013_23836465
M3_4_IFN_Chaussabel2008_18631455
hallmark50_IFNg_response_Liberzon2019_26771021
hallmark50_IFNa_response_Liberzon2019_26771021
M5_12_IFN_Chaussabel2008_18631455
mDC_monocytes_Heise2014_thesis
DDRD_group3_Mulligan2014_24402422
IFN_SLE_Bennett2003_12642603
IFN_MB_Staub2012_22937182
radioresistance_Khodarev2004_14755057
IFN_II_Aybey2025_40611262
eosinophil_Angelova2015_25853550
high_risk_LC_Chen2007_17202451
hallmark50_apical_surface_Liberzon2019_26771021
NOTCH_pathway_breast_Braune2024_38172915
YAP_Mazur2020_aacr2019:A38
typeII_CSC_Lottaz2010_20145155
CIP2_knockdown_up_Niemel2013_22809314
proneural_down_GBM_Verhaak2010_20129251
metastasis_BRCA_lung_Minn2005_16049480
MSL_refined_BRCA_Lehmann2011_21633166
claudin_low_up_BRCA_Prat2013_20813035
CCS3_serrated_CRC_DeSousaEMelo2013_23584090
EMT_BRCA_Lien2008_17603561
stroma_cluster4_BRCA_Farmer2005_15897907
stroma_metagene_BRCA_Farmer2005_19122658
EMT_up_Taube2010_20713713
stem_like_Sadanandam2013_23584089
HC2B_progGroup_GBM_Freije2004_15374961
TGFb_early_Verrecchia2001_11279127
ECM_GBM_Liang2005_15827123
YAP_Wang2019_30380420
hippo_up_YAP_transfection_down_Zhang2008_18413746
mesenchymal_GBM_Phillips2006_16530701
hallmark50_angiogenesis_Liberzon2019_26771021
hallmark50_myogenesis_Liberzon2019_26771021
hallmark50_apical_junction_Liberzon2019_26771021
MED12_KD_MEKi_resistant_Huang2012_23178117
hallmark50_TGFb_Liberzon2019_26771021
hallmark50_uv_response_down_Liberzon2019_26771021
EGFR_Mischel2003_12700671
hallmark50_NOTCH_Liberzon2019_26771021
VGFA_HUVEC_Abe2002_12197474
QMPDA_PCA_Collisson2011_21460848
metastasis_BRCA_bone_Kang2004_12842083
radioresistance_up_BRCA_Speers2016_25904749
PTENi_up_Vivanco2007_17560336
basal_subtype_BRCA_Calza2007_16846532
BL2_refined_BRCA_Lehmann2011_21633166
basal_subtype_LSCC_Wilkerson2011_20643781
basal_like_pdac_Moffit2016_26343385
basal_HNCA_Walter2013_23451093
hallmark50_P53_Liberzon2019_26771021
hallmark50_ER_early_Liberzon2019_26771021
LSCC_vs_LAD_Kuner2009_18486272
NKcell_monocytes_Heise2014_thesis
MTOR_PI3K_S6K_siRNA_Heinonen2008_18652687
neutrophils_Bindea2013_24138885
clusterB_MB_Kool2008_18769486
clusterD_MB_Kool2008_18769486
SHH_MB_Staub2012_22937182
hallmark50_reactive_oxygen_species_Liberzon2019_26771021
classical_subtype_LSCC_Wilkerson2011_20643781
luminal_apocrine_cluster7_BRCA_Farmer2005_15897907
apocrine_basal_hypoxia_cluster3_BRCA_Farmer2005_15897907
hcc_tumorinItiatingEpCAMpos_Yamashita2009_19150350
luminalA_subtype_BRCA_Calza2007_16846532
hallmark50_adipogenesis_Liberzon2019_26771021
hallmark50_faty_acid_metabolism_Liberzon2019_26771021
hallmark50_coagulation_Liberzon2019_26771021
hallmark50_xenobiotic_metabolism_Liberzon2019_26771021
acinar_PDAC_normal_Li2016_26691212
hallmark50_bile_acis_metabolism_Liberzon2019_26771021
enterocyte_Sadanandam2013_23584089
classical_pdac_moffit2016_26343385
TA_Sadanandam2013_23584089
hypoxia_Chi2007_16417408
hallmark50_glycolysis_Liberzon2019_26771021
LAD_vs_LSCC_Kuner2009_18486272
atypical_HNCA_Walter2013_23451093
claudin_low_down_BRCA_Prat2013_20813035
WNT_canonical_KRASdep_Singh2012_22341439
SRC_Bild2006_16273092
hallmark50_WNT_bCatenin_Liberzon2019_26771021
hallmark50_hedgehog_Liberzon2019_26771021
HC1A_progGroup_GBM_Freije2004_15374961
WNT_cluster_MB_Kool2008_18769486
WNT_MB_Staub2012_22937182
clusterE_MB_Kool2008_18769486
beta_PDAC_normal_Li2016_26691212
proNeural_GBM_Phillips2006_16530701
alpha_PDAC_normal_Li2016_26691212
goblet_like_Ragulan2019_31113981
Subtype
SCLC-N
SCLC-A
SCLC-P
SCLC-Y
Signature Score
-2 -1 0 1 2

Supplementary Figure S7. RosettaSX heatmap for TEMPUS SCLC consensus cohort. Heatmap of per-sample signature expression across Tempus SCLC consensus NAPY subtypes (n=383). The per-sample signature score is calculated as the average z-score of the signature genes, for each sample. All 166 signatures displayed in the heatmap demonstrated strong Coherence Scores (CS>0.2), indicating consistent expression changes within the signature gene set across samples, thereby underscoring the relevance of these signatures in our Tempus dataset. All signatures passed our filtering step of differential expression (p-value < 0.05) in at least 3 of the 6 pairwise comparisons by Wilcoxon test.
