# Supplementary material for: Subclassification of Small Cell Lung Cancer Based on Gene Expression Signatures and Machine Learning
Source: Cancer Res Commun. 2026 Mar 12;6(3):545–56. doi: 10.1158/2767-9764.CRC-25-0512 (PMC13012008; doi:10.1158/2767-9764.CRC-25-0512)
Supplement: Supplementary Figure S9 — Selected signatures scored across NAPY subtypes in George cohort. [file crc-25-0512_supplementary_figure_s9_suppsf9.pdf]

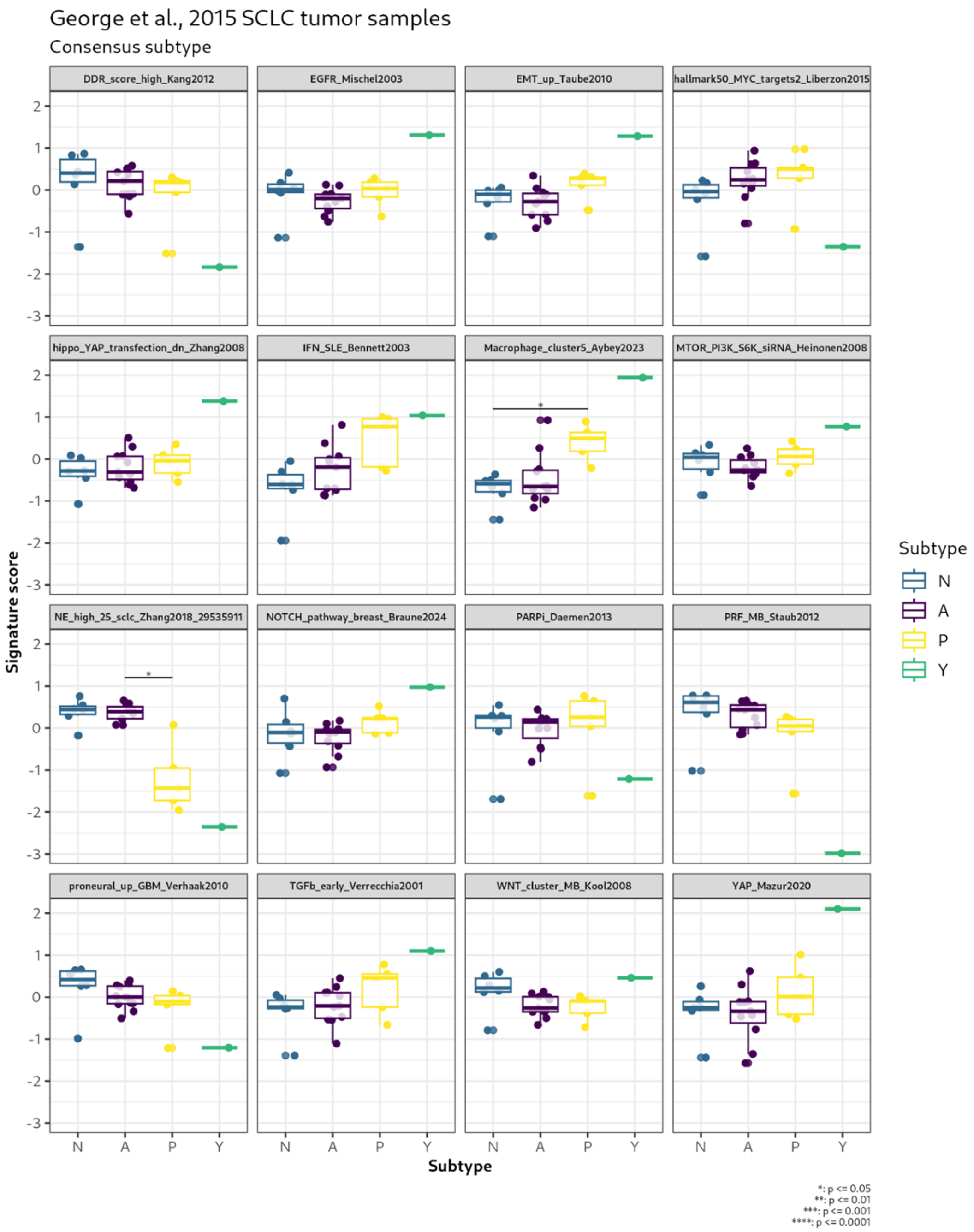

Supplementary Figure S9. Selected signatures scored across NAPY subtypes in George cohort. Boxplot of signature score across George SCLC consensus NAPY subtypes (n=23) for 16 biological pathways that exhibited differential expression across subtypes in the Tempus SCLC cohort. Only samples that demonstrated robust subtype classification according to our proposed strategy: requiring agreement between the predicted class based on the highest z-score TF expression and the predictions made by the SVM NAPY classifier, were considered for the analysis. George et al. SCLC consensus NAPY subtypes group sizes: SCLC-N (n=6), SCLC-A (n=11), SCLC-P (n=5), SCLC-Y (n=1). Although most pairwise comparisons yielded non-significant p-values due to the small sample sizes limiting statistical power, the pathways showed similar trends across subtypes compared to the Tempus cohort.
