# Supplementary material for: Subclassification of Small Cell Lung Cancer Based on Gene Expression Signatures and Machine Learning
Source: Cancer Res Commun. 2026 Mar 12;6(3):545–56. doi: 10.1158/2767-9764.CRC-25-0512 (PMC13012008; doi:10.1158/2767-9764.CRC-25-0512)
Supplement: Supplementary Figure S10 — MYC expression signature versus MYC amplification status. [file crc-25-0512_supplementary_figure_s10_suppsf10.pdf]

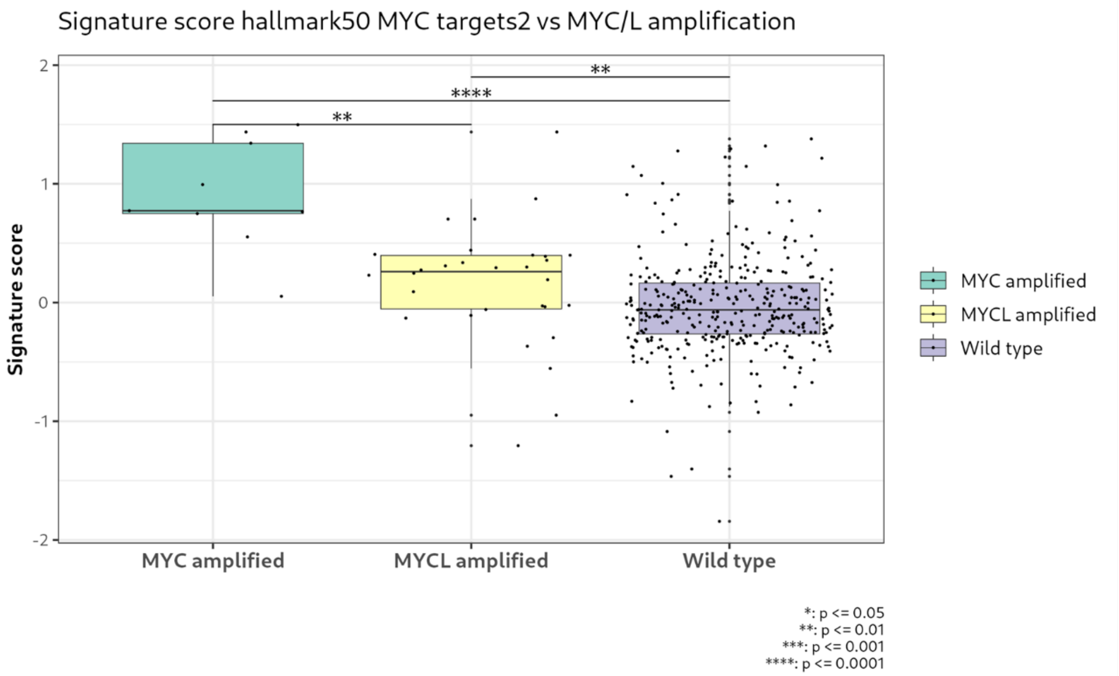

Supplementary Figure S10. MYC expression signature versus MYC amplification status. Boxplot of MYC signature score (hallmark50 targets 2 Liberzon et al. (2015)) against samples with MYC amplified, MYCL amplified and wild type MYC/L in our Tempus SCLC cohort.
