# Supplementary material for: Subclassification of Small Cell Lung Cancer Based on Gene Expression Signatures and Machine Learning
Source: Cancer Res Commun. 2026 Mar 12;6(3):545–56. doi: 10.1158/2767-9764.CRC-25-0512 (PMC13012008; doi:10.1158/2767-9764.CRC-25-0512)
Supplement: Supplementary Figure S11 — Survival characteristics of SCLC NAPY subtypes [file crc-25-0512_supplementary_figure_s11_suppsf11.pdf]

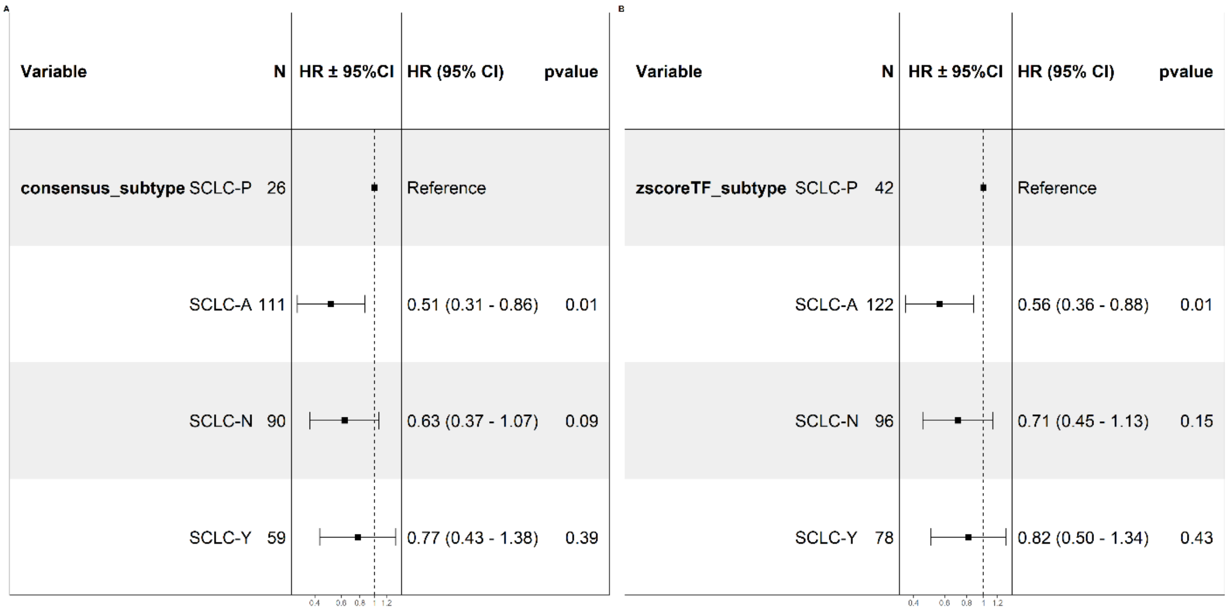

Supplementary Figure S11. Survival characteristics of SCLC NAPY subtypes. A. Univariate Cox Proportional-Hazards evaluating the effect of molecular subtype in survival, in Stage IV Tempus SCLC patients, with molecular subtypes defined using our consensus approach integrating both TF-based and ML/signature-based methods. HRs are relative to SCLC-P, with 95% CI and statistical significance measured with Wald test p-value. **B.** Univariate Cox Proportional-Hazards evaluating the effect of molecular subtype in survival, in Stage IV Tempus SCLC patients, with molecular subtypes defined by the highest z-score TF-expression. HRs are relative to SCLC-P, with 95% CI and statistical significance measured with Wald test p-value. Notably, when molecular subtypes are defined based on our consensus approach, the observed survival HRs are further from 1, and the statistical p-values are lower, highlighting the increased capability to detect differential signals in overall survival.
