# Supplementary material for: Subclassification of Small Cell Lung Cancer Based on Gene Expression Signatures and Machine Learning
Source: Cancer Res Commun. 2026 Mar 12;6(3):545–56. doi: 10.1158/2767-9764.CRC-25-0512 (PMC13012008; doi:10.1158/2767-9764.CRC-25-0512)
Supplement: Supplementary Figure S12 — MYC hallmark expression signature across NAPY subtypes [file crc-25-0512_supplementary_figure_s12_suppsf12.pdf]

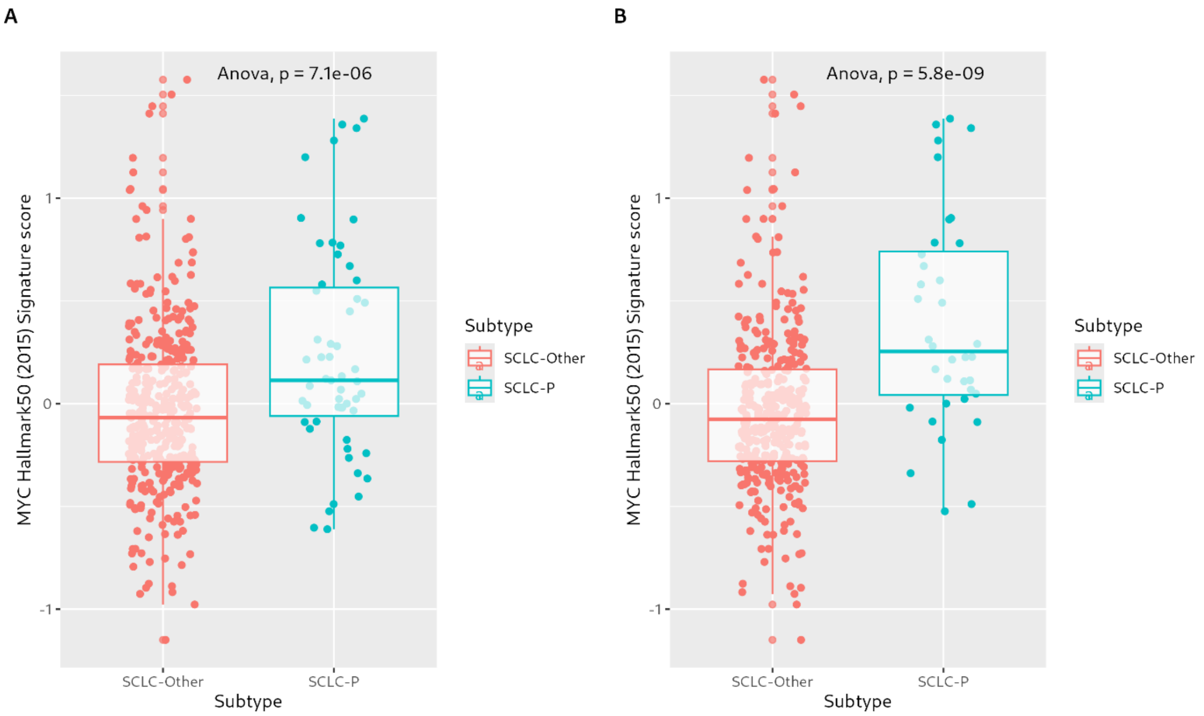

Supplementary Figure S12. MYC hallmark expression signature across NAPY subtypes. Boxplot comparing the difference on the MYC hallmark signature (Liberzon et al., 2015) between the consensus SCLC-P and the other SCLC subtypes under two scenarios: (A) when subtypes are assigned solely based on the TF-expression (left), (B) when subtypes are assigned based the agreement of both the TF-based and our ML/signature-based derived class (right). Notably, the second scenario increases the capability to detect differential signals in pathway activity among the SCLC groups, reducing the p-value of ANOVA statistical test from 7.1e-6 to 5.8e-09.
