# Supplementary material for: Subclassification of Small Cell Lung Cancer Based on Gene Expression Signatures and Machine Learning
Source: Cancer Res Commun. 2026 Mar 12;6(3):545–56. doi: 10.1158/2767-9764.CRC-25-0512 (PMC13012008; doi:10.1158/2767-9764.CRC-25-0512)
Supplement: Supplementary Table S1 — NAPY labels for independent validation. [file crc-25-0512_supplementary_table_s1_suppst1.pdf]

Name	Type of sample	Source	Subtype assignment
COLO-668	Cell line	CCLE	SCLC-A
COR-L47	Cell line	CCLE	SCLC-A
COR-L88	Cell line	CCLE	SCLC-A
COR-L95	Cell line	CCLE	SCLC-A
DMS-153	Cell line	CCLE	SCLC-A
DMS-454	Cell line	CCLE	SCLC-A
DMS-53	Cell line	CCLE	SCLC-A
DMS-79	Cell line	CCLE	SCLC-A
NCI-H1092	Cell line	CCLE	SCLC-A
NCI-H1105	Cell line	CCLE	SCLC-A
NCI-H1184	Cell line	CCLE	SCLC-A
NCI-H1436	Cell line	CCLE	SCLC-A
NCI-H146	Cell line	CCLE	SCLC-A
NCI-H1618	Cell line	CCLE	SCLC-A
NCI-H1836	Cell line	CCLE	SCLC-A
NCI-H1876	Cell line	CCLE	SCLC-A
NCI-H1930	Cell line	CCLE	SCLC-A
NCI-H1963	Cell line	CCLE	SCLC-A
NCI-H2029	Cell line	CCLE	SCLC-A
NCI-H2081	Cell line	CCLE	SCLC-A
NCI-H209	Cell line	CCLE	SCLC-A
NCI-H2196	Cell line	CCLE	SCLC-A
NCI-H510	Cell line	CCLE	SCLC-A
NCI-H69	Cell line	CCLE	SCLC-A
NCI-H889	Cell line	CCLE	SCLC-A
SHP-77	Cell line	CCLE	SCLC-A
COR-L24	Cell line	CCLE	SCLC-N
COR-L279	Cell line	CCLE	SCLC-N
DMS-273	Cell line	CCLE	SCLC-N
HCC-33	Cell line	CCLE	SCLC-N
NCI-H1694	Cell line	CCLE	SCLC-N
NCI-H2171	Cell line	CCLE	SCLC-N
NCI-H2227	Cell line	CCLE	SCLC-N
NCI-H446	Cell line	CCLE	SCLC-N
NCI-H524	Cell line	CCLE	SCLC-N
NCI-H82	Cell line	CCLE	SCLC-N
SCLC-21H	Cell line	CCLE	SCLC-N
COR-L311	Cell line	CCLE	SCLC-P
NCI-H1048	Cell line	CCLE	SCLC-P
NCI-H211	Cell line	CCLE	SCLC-P
NCI-H526	Cell line	CCLE	SCLC-P
DMS-114	Cell line	CCLE	SCLC-Y
NCI-H1341	Cell line	CCLE	SCLC-Y
NCI-H196	Cell line	CCLE	SCLC-Y
NCI-H2286	Cell line	CCLE	SCLC-Y
NCI-H841	Cell line	CCLE	SCLC-Y
SBC-5	Cell line	CCLE	SCLC-Y
SW-1271	Cell line	CCLE	SCLC-Y
S00022	Tumour	George (2015)	SCLC-A

Name	Type of sample	Source	Subtype assignment
S00035T	Tumour	George (2015)	SCLC-A
S00825	Tumour	George (2015)	SCLC-A
S00832	Tumour	George (2015)	SCLC-A
S00838	Tumour	George (2015)	SCLC-A
S01297T	Tumour	George (2015)	SCLC-A
S01366	Tumour	George (2015)	SCLC-A
S01512	Tumour	George (2015)	SCLC-A
S01524	Tumour	George (2015)	SCLC-A
S01578	Tumour	George (2015)	SCLC-A
S01861T	Tumour	George (2015)	SCLC-A
S01864	Tumour	George (2015)	SCLC-A
S02065	Tumour	George (2015)	SCLC-A
S02120	Tumour	George (2015)	SCLC-A
S02194	Tumour	George (2015)	SCLC-A
S02241	Tumour	George (2015)	SCLC-A
S02242	Tumour	George (2015)	SCLC-A
S02243	Tumour	George (2015)	SCLC-A
S02244	Tumour	George (2015)	SCLC-A
S02248	Tumour	George (2015)	SCLC-A
S02249	Tumour	George (2015)	SCLC-A
S02284	Tumour	George (2015)	SCLC-A
S02285	Tumour	George (2015)	SCLC-A
S02287	Tumour	George (2015)	SCLC-A
S02289	Tumour	George (2015)	SCLC-A
S02290	Tumour	George (2015)	SCLC-A
S02291	Tumour	George (2015)	SCLC-A
S02295	Tumour	George (2015)	SCLC-A
S02297	Tumour	George (2015)	SCLC-A
S02328	Tumour	George (2015)	SCLC-A
S02376T	Tumour	George (2015)	SCLC-A
S02378T	Tumour	George (2015)	SCLC-A
S02382T	Tumour	George (2015)	SCLC-A
S00831	Tumour	George (2015)	SCLC-A
S02322	Tumour	George (2015)	SCLC-A
S02360	Tumour	George (2015)	SCLC-A
S02397	Tumour	George (2015)	SCLC-A
S01873T	Tumour	George (2015)	SCLC-N
S02139	Tumour	George (2015)	SCLC-N
S02293	Tumour	George (2015)	SCLC-N
S02294	Tumour	George (2015)	SCLC-N
S00829	Tumour	George (2015)	SCLC-P
S02209	Tumour	George (2015)	SCLC-P
S02256	Tumour	George (2015)	SCLC-P
S02286	Tumour	George (2015)	SCLC-P
S02296	Tumour	George (2015)	SCLC-P
S02375T	Tumour	George (2015)	SCLC-P
S02246	Tumour	George (2015)	SCLC-Y

Supplementary Table S1. NAPY labels for independent validation. Samples used for independent validation of SVM classifier. CCLE cell lines, publicly available in DepMapPortal. George *et al*., 2015 tumor samples publicly available from BioPortal. Labels taken from Rudin *et al.* 2019 Supplementary Data.
