# Supplementary material for: Subclassification of Small Cell Lung Cancer Based on Gene Expression Signatures and Machine Learning
Source: Cancer Res Commun. 2026 Mar 12;6(3):545–56. doi: 10.1158/2767-9764.CRC-25-0512 (PMC13012008; doi:10.1158/2767-9764.CRC-25-0512)
Supplement: Supplementary Table S2 — Per-class classification performance on external data [file crc-25-0512_supplementary_table_s2_suppst2.pdf]

	Assessment on 20% Tempus spared out records (n=68)				*Further validation on CCLE cell lines* (n=48)				*Further validation on George et al tumors (n=48)*			
	**A**	**N**	**P**	**Y**	**A**	**N**	**P**	**Y**	**A**	**N**	**P**	**Y**
accuracy	0.93	0.96	0.97	0.94	0.96	0.96	0.94	0.98	0.79	0.90	0.92	0.98
bal accy	0.93	0.95	0.89	0.89	0.96	0.94	0.97	0.93	0.86	0.94	0.95	0.99
Sens	0.96	0.95	0.78	0.80	0.92	0.91	1.00	0.86	0.73	1.00	1.00	1.00
Spec	0.91	0.96	1.00	0.98	1.00	0.97	0.93	1.00	1.00	0.89	0.90	0.98
precision	0.85	0.91	1.00	0.92	1.00	0.91	0.57	1.00	1.00	0.44	0.60	0.50
recall	0.96	0.95	0.78	0.80	0.92	0.91	1.00	0.86	0.73	1.00	1.00	1.00
f_meas	0.90	0.93	0.88	0.86	0.96	0.91	0.73	0.92	0.84	0.62	0.75	0.67
Npv	0.98	0.98	0.97	0.95	0.92	0.97	1.00	0.98	0.52	1.00	1.00	1.00
Ppv	0.85	0.91	1.00	0.92	1.00	0.91	0.57	1.00	1.00	0.44	0.60	0.50
Final NAPY SVM Classifier, cost=0.031, features=80 genes												

Supplementary Table S2. Per-class classification performance on external data. Per-class classification performance results for the reserved 20% of Tempus records, for the CCLE dataset and for the George et al. dataset, measured as one-vs-rest binary classification. Associated confusion matrices are presented in Supplementary Table S3, Supplementary Table S4 and Supplementary Table S5.
