# Supplementary material for: Subclassification of Small Cell Lung Cancer Based on Gene Expression Signatures and Machine Learning
Source: Cancer Res Commun. 2026 Mar 12;6(3):545–56. doi: 10.1158/2767-9764.CRC-25-0512 (PMC13012008; doi:10.1158/2767-9764.CRC-25-0512)
Supplement: Supplementary Table S3 — Confusion matrix TEMPUS hold-out data. [file crc-25-0512_supplementary_table_s3_suppst3.pdf]

Confusion Matrix - Tempus 20% records saved for assessment					
		TRUTH CLASS			
		A	N	P	Y
PREDICTED CLASS	A	**22**	1	0	3
	N	1	**20**	1	0
	P	0	0	**7**	0
	Y	0	0	1	**12**

Supplementary Table S3. Confusion matrix TEMPUS hold-out data. Confusion matrix derived from predictions for 20% Tempus SCLC records saved for assessment (n=68) of the final NAPY SVM classifier.
