# Supplementary material for: Subclassification of Small Cell Lung Cancer Based on Gene Expression Signatures and Machine Learning
Source: Cancer Res Commun. 2026 Mar 12;6(3):545–56. doi: 10.1158/2767-9764.CRC-25-0512 (PMC13012008; doi:10.1158/2767-9764.CRC-25-0512)
Supplement: Supplementary Table S4 — Confusion matrix CCLE cell lines. [file crc-25-0512_supplementary_table_s4_suppst4.pdf]

Confusion Matrix - CCLE cell lines					
		TRUTH CLASS			
		A	N	P	Y
PREDICTED CLASS	A	**24**	0	0	0
	N	1	**10**	0	0
	P	1	1	**4**	1
	Y	0	0	0	**6**

Supplementary Table S4. Confusion matrix CCLE cell lines. Confusion matrix derived from prediction of CCLE cell lines (n=48) with final NAPY SVM classifier.
