# Supplementary material for: Subclassification of Small Cell Lung Cancer Based on Gene Expression Signatures and Machine Learning
Source: Cancer Res Commun. 2026 Mar 12;6(3):545–56. doi: 10.1158/2767-9764.CRC-25-0512 (PMC13012008; doi:10.1158/2767-9764.CRC-25-0512)
Supplement: Supplementary Table S5 — Confusion matrix George cohort. [file crc-25-0512_supplementary_table_s5_suppst5.pdf]

Confusion Matrix – George et al., tumors					
		TRUTH CLASS			
		A	N	P	Y
PREDICTED CLASS	A	**27**	0	0	0
	N	5	**4**	0	0
	P	4	0	**6**	0
	Y	1	0	0	**1**

Supplementary Table S5. Confusion matrix George cohort. Confusion matrix derived from prediction of George *et al.* tumors (n=48) with final NAPY SVM classifier.
