# Supplementary material for: Subclassification of Small Cell Lung Cancer Based on Gene Expression Signatures and Machine Learning
Source: Cancer Res Commun. 2026 Mar 12;6(3):545–56. doi: 10.1158/2767-9764.CRC-25-0512 (PMC13012008; doi:10.1158/2767-9764.CRC-25-0512)
Supplement: Supplementary Table S6 — Literature evidence of TF regulation for the four 20-gene NAPY gene expression signatures. [file crc-25-0512_supplementary_table_s6_suppst6.pdf]

Signature	Gene	Reference-PMID	ChIP-Atlas
ASCL1 associated	*SEC11C*	34653364, 25267614, 35263037	yes
	DDC	34466783, 27452466, 29945888	yes
	RIMKLA	34653364, 35263037	yes
	CNKSR3	35477723	yes
	SCN3A	34653364, 25267614	yes
	PTPRN2	34653364, 25267614, 35263037	yes
	CACNA1A	27452466, 25267614	yes
	RGS17	34653364, 35263037	yes
	STK32A	35263037	yes
	GRP	34653364, 27452466, 29945888, 36195615, 25267614, 35263037	yes
	CA8	34653364	No
	NOL4	34653364, 35263037	yes
	RAB3B	29132337, 25267614	yes
	SMPD3	34653364, 35477723, 35263037	yes
	FBLN7	34653364, 35263037	yes
	MS4A8	29945888, 32170367, 35981544	yes
	ETS2	34653364, 25267614, 35263037	yes
	DLL3	27452466, 37062002, 33482121, 30926931, 35263037	yes
	SLC36A4	34653364, 25267614, 35263037	yes
	NR0B2	35981544, 25267614, 35263037	yes
NEUROD1 associated	*CERKL*	35981544	yes
	SSTR2	27452466, 33482121, 35263037	yes
	CHRNB4	27452466, 35263037	yes
	CHRNA3	27452466, 36195615	yes
	NHLH1	27452466, 32473656, 35263037	yes
	NEUROD4	34466783, 27452466, 32473656	yes
	LMO1	34653364	yes
	PPP1R17	35981544	yes
	NEUROD2	27452466, 35981544, 35263037	yes
	CNTN2	36195615, 35263037	yes
	SLC17A6	34653364	yes
	FNDC5	34653364, 35263037	yes
	SHF	34653364	yes
	THSD7B	34653364	yes
	DACH1	34653364	yes
	HPCA	34653364, 27452466, 35263037	yes
	PROKR1	34653364	yes
	GNG8	34653364	yes
	KIAA1614	34653364, 35263037	yes
	CLVS1	34653364	yes
POU2F3 associated	*C11orf53*	36197978	yes
	GFI1B	30926931, 35569741	yes
	TRPM5	33718595	yes
	APOBEC1	34653364	yes
	FAM150A	39605338	No
	IL19	34653364	yes
	PTPN18	34653364	yes
	HES2		No
	CALML5	29945888	No
	IMP4	34653364	yes
	BMX	35981544	yes
	ART3	34653364	yes
	LANCL3	34653364	yes
	PVRL4 (NECTIN4)	39605338	No
	KLHDC7A	34653364	yes
	CALHM3	34653364	yes
	KIAA1024L (MINAR2)	33086069	No
	ADAMTS19	34653364	yes
	ASCL2	35569741, 29535911	yes
	COLCA2	34653364	yes
YAP1 associated	*LATS2*	35345340, 32721553	yes
	WWTR1	34653364	yes
	LAMB2	34653364	yes
	OSMR	34466783, 32721553, 35121965	yes
	CYBRD1	34653364	yes
	GPX8	34653364, 35981544, 35121965	No
	MSRB3	34653364, 35981544	yes
	ITGB5	34653364	yes
	SYDE1	34653364	yes
	RBMS3	32721553, 35121965	yes
	PLA2R1	34653364	yes
	WTIP	34653364	yes
	EPHA2	30926931, 29535911, 32721553, 35121965	yes
	MYL9	34653364, 35981544, 32721553	yes
	PMP22	34653364	yes
	ZCCHC24	34653364	yes
	EHD2	34653364	yes
	IFITM3	35981544, 33086069, 29535911, 35121965	yes
	AXL	33482121, 30926931, 32721553, 37455012	yes
	THBS1	30055645	yes

Supplementary Table S6. Literature evidence of TF regulation for the four 20-gene NAPY gene expression signatures. The vast majority of genes in NAPY signatures have been described as bound by NAPY TFs in ChIP omics databases. References describing these genes in the context of SCLC are included: PMID, ChiP-Atlas (https://chip-atlas.org).
