# Supplementary material for: Subclassification of Small Cell Lung Cancer Based on Gene Expression Signatures and Machine Learning
Source: Cancer Res Commun. 2026 Mar 12;6(3):545–56. doi: 10.1158/2767-9764.CRC-25-0512 (PMC13012008; doi:10.1158/2767-9764.CRC-25-0512)
Supplement: Supplementary Table S7 — Confusion matrix TF-based vs ML-based NAPY classification. [file crc-25-0512_supplementary_table_s7_suppst7.pdf]

Confusion Matrix – SCLC all samples (n=460)					
		TF-class (strongest z-score TF)			
		A	N	P	Y
ML-class (expression signature)	A	**150**	14	8	15
	N	8	**118**	8	7
	P	0	2	**38**	3
	Y	7	2	3	**79**

Supplementary Table S7. Confusion matrix TF-based vs ML-based NAPY classification. Confusion matrix of Tempus SCLC cohort (n=460) comparing the predicted class labels of both the TF-based and ML/signature-based methods. For 383 of 460 SCLCs we obtained a consensus between the methods. The distribution of consensus subtype samples (n=383) was 150 SCLC-A (39%), 118 SCLC-N (31%), 36 SCLC-P (9%), and 79 SCLC-Y (21%). These consensus samples have been used for subsequent analysis of patterns across NAPY subtypes.
