# Supplementary material for: Subclassification of Small Cell Lung Cancer Based on Gene Expression Signatures and Machine Learning
Source: Cancer Res Commun. 2026 Mar 12;6(3):545–56. doi: 10.1158/2767-9764.CRC-25-0512 (PMC13012008; doi:10.1158/2767-9764.CRC-25-0512)
Supplement: Supplementary Table S8 — Univariate survival analysis in Stage IV TEMPUS patients. [file crc-25-0512_supplementary_table_s8_suppst8.pdf]

Variable	Level	HR (95% CI for HR)	p.value
**Tempus SCLC stage IV patients**			
**Consensus subtype**	SCLC-P	Reference	**-**
	SCLC-A	0.51 (0.31-0.86)	**0.01**
	SCLC-N	0.63 (0.37-1.1)	0.09
	SCLC-Y	0.77 (0.43-1.4)	0.39
**Sex**	Female	Reference	-
	Male	1.2 (0.91-1.7)	0.17
**Age**	-	1.0 (1-1)	0.07
**T effector score**	-	0.77 (0.63-0.93)	**0.01**
**Tumor-associated macrophage score**	-	1.2 (0.95-1.5)	0.12
**Tempus SCLC stage IV patients, receiving Chemo+ICI**			
**Consensus subtype**	SCLC-P	Reference	**-**
	SCLC-A	0.51 (0.25-1)	**0.05**
	SCLC-N	0.45 (0.22-0.92)	**0.03**
	SCLC-Y	0.59 (0.26-1.3)	0.19
**Sex**	Female	Reference	**-**
	Male	1.8 (1.1-3)	**0.01**
**Age**	-	1 (1-1)	0.10
**T effector score**	-	0.78 (0.61-1)	**0.06**
**Tumor-associated macrophage score**	-	0.98 (0.72-1.3)	0.89

Supplementary Table S8. Univariate survival analysis in Stage IV TEMPUS patients. Univariate Cox Proportional-Hazards model results, evaluating the effect of independent covariates on survival, for Tempus SCLC stage IV patients, and for Tempus SCLC stage IV patients receiving Chemo+ICI (n=127). Statistical significance has been assessed using the Wald test and resulting p-values are included.
