# Supplementary material for: Subclassification of Small Cell Lung Cancer Based on Gene Expression Signatures and Machine Learning
Source: Cancer Res Commun. 2026 Mar 12;6(3):545–56. doi: 10.1158/2767-9764.CRC-25-0512 (PMC13012008; doi:10.1158/2767-9764.CRC-25-0512)
Supplement: Supplementary Figure S6 — Selected signatures scored across NAPY subtypes in TEMPUS cohort. [file crc-25-0512_supplementary_figure_s6_suppsf6.pdf]

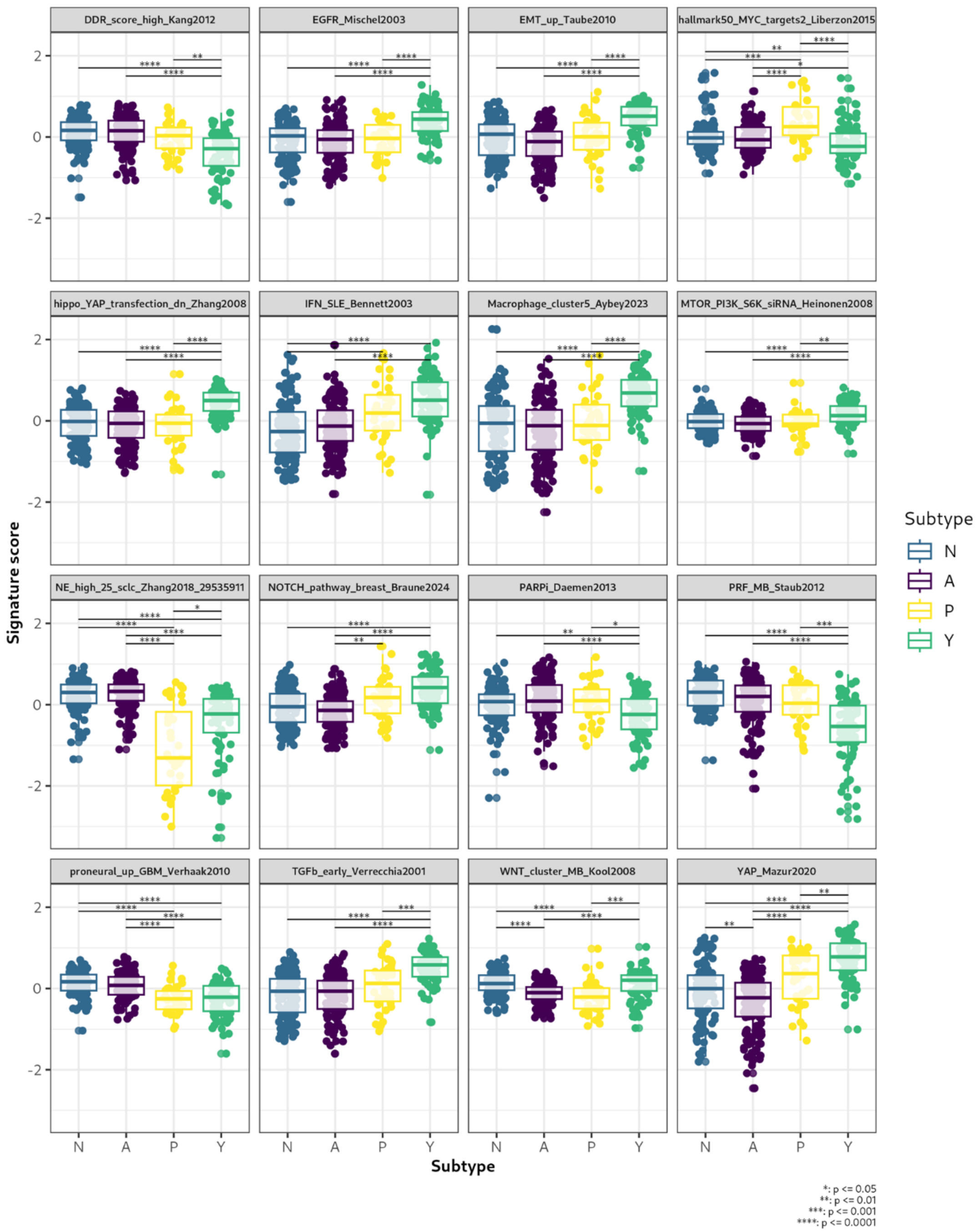

Supplementary Figure S6. Selected signatures scored across NAPY subtypes in TEMPUS cohort. Boxplot of signature score across Tempus SCLC consensus NAPY subtypes (n=383) for 16 relevant biological pathways from Heatmap. All 16 signatures displayed in the boxplot demonstrated strong Coherence Scores (CS>0.2), indicating consistent expression changes within the signature gene set across samples, thereby underscoring the relevance of these signatures in our Tempus dataset (n=383). All signatures passed our filtering step of differential expression (p-value < 0.05) in at least 3 of the 6 pairwise comparisons by Wilcoxon test. Adjusted p-value of pairwise differential expression across subtypes included as annotation.
